# SPOCK2-mediated phenotype switch of vascular smooth muscle cells contributes to aortic aneurysm in Kawasaki disease

**DOI:** 10.3389/fimmu.2026.1902009

**Published:** 2026-10-23

**Authors:** Xiao Xu, Yucong Wei, Min Wang, Jiahui Liu, Ting Zhao, Xian Ma, Tielong Chen, Buddhadeb Dawn, Fangqi Gong, Lin Zhao

**Affiliations:** 1 Department of Cardiology, Hangzhou TCM Hospital Affiliated to Zhejiang Chinese Medical University, Hangzhou, Zhejiang, China; 2 Department of Cardiology, Children’s Hospital, Zhejiang University School of Medicine, National Clinical Research Center for Children and Adolescents’ Health and Diseases, Hangzhou, Zhejiang, China; 3 School of Basic Medical Sciences and Forensic Medicine, Hangzhou Medical College, Hangzhou, Zhejiang, China; 4 Department of Gynecology, Hangzhou TCM Hospital Affiliated to Zhejiang Chinese Medical University, Hangzhou, Zhejiang, China; 5 Department of Internal Medicine, Kirk Kerkorian School of Medicine at the University of Nevada, Las Vegas, NV, United States

**Keywords:** aneurysm, Kawasaki disease, MMP2, phenotype switch, SPOCK2, VSMC

## Abstract

**Background:**

Coronary artery aneurysm is a major cardiovascular complication of Kawasaki disease (KD). The phenotype switch of vascular smooth muscle cells (VSMCs) plays a crucial role in pathological vascular wall remodeling in KD; however, the molecular mechanisms driving this process remain incompletely understood. Although the SPARC protein family has been implicated in various vascular diseases, the role of specific SPARC family members in mediating VSMC phenotypic transition during KD-associated vasculitis remains unknown. Here, we investigated the function and mechanistic role of SPOCK2, a SPARC family member, in vascular wall homeostasis under KD-induced inflammatory stress.

**Methods:**

SPOCK2 transcript expression was evaluated in public transcriptomic datasets and clinical KD cohorts. To model KD vasculitis *in vivo*, mice were challenged with *Candida albicans* water-soluble fraction (CAWS). Adeno-associated virus (AAV)2/9-mediated gene transfer was employed for targeted in vivo overexpression of SPOCK2 in mouse aortas. VSMC phenotypic transition, vascular remodeling, extracellular matrix (ECM) integrity, and local cytokine production were assessed using immunofluorescence, RNA sequencing, quantitative RT-PCR, and co-immunoprecipitation assays to delineate SPOCK2–MMP2 structural and functional interactions.

**Results:**

Transcriptional profiling of clinical KD cohorts revealed that *SPOCK2* is consistently downregulated in the acute phase of the disease. In murine KD vasculitis models, SPOCK2 expression was notably depleted in both aortic root and abdominal aorta, correlating with severe vascular inflammation and aneurysm formation. Targeted in vivo overexpression of SPOCK2 via AAV2/9 effectively restored the contractile VSMC phenotype, thereby attenuating vasculitis and preventing abdominal aortic aneurysm formation. Mechanistically, SPOCK2 acts as a specific endogenous inhibitor of MMP2. Through physical interaction, SPOCK2 binds to MMP2, hindering its enzymatic activation. In the inflammatory microenvironment of KD, the pathological loss of SPOCK2 leads to unchecked MMP2 activity, promoting uncontrolled extracellular matrix degradation and pathogenic contractile-to-synthetic transition of VSMCs.

**Conclusion:**

Our findings establish SPOCK2 as a novel protective factor in vascular wall homeostasis and a potential candidate for preventive strategies targeting vascular remodeling and systemic vasculitis in Kawasaki disease.

## Introduction

Kawasaki disease (KD), also known as mucocutaneous lymph node syndrome, is an acute, self-limiting systemic vasculitis that primarily affects children under the age of five, with a higher incidence in boys (1). Although both genetic and environmental factors contribute to the susceptibility to KD, the etiology of KD remains incompletely understood. The most severe complication of KD is the development of coronary artery aneurysms, which occurs in approximately 20%-25% of untreated KD patients (2).

The pathophysiologic mechanisms underlying the formation of coronary artery aneurysm in KD likely involve multifaceted molecular processes leading to pathological remodeling of the blood vessel wall (3). Vascular smooth muscle cells (VSMCs) and extracellular matrix (ECM) are main components of the media layer of blood vessels, and act as primary contributors to vascular remodeling (4). VSMCs are notable for phenotypic plasticity, which enables them to display two main phenotypes, the contractile and the synthetic, in response to diverse vascular microenvironments (5). Typically, most mature VSMCs exhibit a contractile phenotype, characterized by elevated contractile protein markers to maintain vascular tone and function. In pathological environments, contractile VSMCs often transition into a synthetic phenotype, which is considered a key event in arterial remodeling (6). Synthetic VSMCs are characterized by reduced expression of contractile proteins and increased expression of synthetic markers and ECM-related proteins (7). However, the precise role of VSMC phenotype switch in the intense inflammatory microenvironment of KD vasculitis has not been elucidated.

SPARC (secreted protein acidic and rich in cysteine) protein family comprises a group of matricellular proteins, including SPARC, SPARCL1, FSTL-1, SMOC-1, SMOC-2, SPOCK1, SPOCK2, and SPOCK3, which serve as critical regulators of vascular homeostasis and are essential for maintaining the integrity of the extracellular matrix (8, 9). Recently, increasing evidence has shown that SPARC protein family members participate in the process of VSMC phenotype switch during aneurysm formation. SPARC has been demonstrated to be significantly expressed in VSMCs within intracranial aneurysms, which promoted VSMC synthetic phenotype transition by inhibiting contractile gene expression (10). Moreover, SPOCK2 was upregulated in synthetic aortic smooth muscle cells in sporadic non-syndromic thoracic aortic aneurysms (SNSTAA), and the plasma levels of secreted SPOCK2 represent an early marker of a developing SNSTAA (11). However, whether the SPARC protein family is involved in the development of KD vasculitis remains unknown.

Accordingly, in the current study, we elucidated the pathological role and molecular mechanism of SPOCK2 in KD-associated aneurysms. We investigated the transformation of VSMCs to a fibroblast-like phenotype during KD vasculitis and identified that SPOCK2 is a key regulator of this phenotype switch, participating in the progression of aortic aneurysm in KD. Additionally, we report that SPOCK2 interacts with matrix metallopeptidase 2 (MMP2) to dampen the activation of MMP2, thereby maintaining the contractile phenotype of VSMCs and alleviating *Candida albicans* water-soluble fraction (CAWS)-induced abdominal aortic aneurysms. These findings elucidate an important protective role for SPOCK2 in the pathogenesis of vascular lesions in KD and provide valuable insights for future preventive and protective strategies.

## Materials and methods

We conducted this study in accordance with the principles of the Declaration of Helsinki. The protocols for collecting peripheral blood from human subjects were approved by the Ethics Committee of Children’s Hospital, Zhejiang University School of Medicine (No. 2021-IRB-320). The animal experiments in the present study complied with the ARRIVE guidelines. They were conducted in accordance with U.S. Public Health Service Policy on the Humane Care and Use of Laboratory Animals. All animal experiment protocols were approved by the Animal Care and Use Committee of the Zhejiang Chinese Medical University (IACUC-20230417-15).

### Human peripheral blood collection

The parents of study participants provided informed consent before blood collection. The patients enrolled in the KD group (n = 16) were diagnosed based on KD diagnostic guidelines from the American Heart Association. Peripheral blood samples were collected from KD patients at the acute phase before the initial IVIG treatment. The control group consisted of 6 healthy children, with a comparable age and gender distribution to the KD group. These enrolled KD patients and healthy controls were recruited between 2022 and 2023 at Children’s Hospital, Zhejiang University School of Medicine. All human peripheral blood samples were stored at -80 °C.

### Mice

Four-week-old male C57BL/6 mice were obtained from GemPharmatech (Nanjing, Jiangsu, China). Mice were housed under specific pathogen-free conditions at a temperature of 22-24 °C with a standard light-dark cycle (12:12). Mice had free access to chow diet and water. Only male mice were used in this study as CAWS-injection induces stronger and more consistent coronary vasculitis lesions and abdominal aortic aneurysms in males compared with females.

### Preparation of Candida Albicans Water-Soluble Fraction

The CAWS was prepared by *Candida albicans* (NBRC 1385) (ATCC, VA, USA) following the protocol reported by Stock et al. (12), with minor modifications. Briefly, *Candida albicans* were cultured in a C-limiting medium at 27 °C for 48 hours. To collect the CAWS, an equal volume of ethanol was added to the culture and incubated overnight at 4 °C. Subsequently, the precipitate was suspended in an aliquot of double-distilled water. After centrifugation, the water-soluble fraction was mixed with an equal volume of ethanol and left overnight at 4 °C. This solution underwent another round of centrifugation, followed by drying of the pellet in acetone overnight. Finally, the CAWS complex was dissolved in PBS and autoclaved before use.

### CAWS-induced vasculitis and AAV2/9-SPOCK2 Injection in mice

Mice were randomly assigned to groups. For induction of vasculitis, mice received intraperitoneal injections of CAWS (4mg/mouse/day) for five consecutive days. Mice in the control group received intraperitoneal injections of an equivalent volume of phosphate-buffered saline (PBS). On day 28 after the last CAWS injection, mice were anesthetized and subsequently euthanized. The abdominal aorta was exposed and imaged, and its maximal diameter was determined by measuring five different regions using ImageJ software. Both aortic root and abdominal aorta were excised for subsequent experiments.

Adeno-associated virus serotype 2/9 (AAV-2/9) vectors encoding *Spock2* (pAAV-CMV-SPOCK2-HA-EF1-GdGreen-WPRE, AAV2/9*-*SPOCK2) and control vectors (pAAV-CMV-MCS-EF1-GdGreen-WPRE, AAV2/9-Vector) were purchased from OBIO Technology (Shanghai, China). Mice received either AAV2/9-vector or AAV2/9-SPOCK2 injection one week prior to the initiation of CAWS injection. Each mouse was administered 1.0 x 10^11^ viral genomes (vg) of AAV2/9 in 200 μL of sterile PBS via the tail vein injection.

### Tissue preparations

Mice were euthanized via isoflurane inhalation, and peripheral blood was collected by cardiac puncture. Subsequently, mice were transcardially perfused with PBS to remove residual blood. Abdominal aortas were photographed under microscope; the maximal abdominal aorta diameter was measured at 5 different areas with ImageJ (13). Abdominal aortic aneurysm was quantitatively defined as > 50% of the maximal outer diameter of the abdominal aorta relative to the mean diameter of age-matched control mice (14). Hearts and abdominal aortas were then harvested. For histological staining, hearts were sliced into three sections from apex to base using a heart slicing matrix. Thereafter, hearts and abdominal aortas were fixed in 5% paraformaldehyde solution. For molecular analysis, both aortic root and abdominal aorta were excised and snap-frozen in liquid nitrogen before being stored at -80 °C for subsequent experiments.

### Histological staining

Paraffin-embedded tissue blocks were sectioned at 5 μm thickness and subjected to Hematoxylin-eosin (H&E), Masson’s Trichrome staining and Verhoeff-van Gieson (EVG) staining. Aortic root sections, which included the secondary coronary artery branch separating from the aorta, were selected for evaluating inflammatory cell infiltration. The severity of inflammatory infiltration was graded using a scoring system reflecting heart vessel inflammation (13). To quantify elastic fiber integrity within the aortic wall, cross-sections stained with EVG were imaged at high magnification under identical illumination settings. Image analysis was conducted using ImageJ software (NIH, Bethesda, MD, USA). Briefly, the tunica media region bounded by internal and external elastic laminae was manually defined as the region of interest (ROI). Color thresholding was applied specifically to isolate the dark purple/black-stained elastic fibers from the surrounding background tissue. The area occupied by the thresholded elastic fibers within the media layer was measured and normalized to the total area of the tunica media ROI. Results are presented as the percentage of elastic fiber area in the media layer. All assessments were performed by investigators in a blinded manner.

### Immunostaining

Paraffinized sections were subjected to immunofluorescence staining. Slides were incubated with the primary antibody against α-SMA (Cell Signaling Technology, Cat# 19245) and SPOCK2 (Santa Cruz Technology, Cat# sc-515691). Following three washes with PBS, sections were incubated with the secondary antibody. Slides were mounted using an anti-fade mounting medium (Beyotime, Cat# P0126). Fluorescence images were captured using a Leica SP8 confocal microscope. All images across both groups were acquired using identical exposure times, gain settings, and laser intensities. Semi-quantification of the fluorescence signal was performed using Image J software (NIH, Bethesda, USA) on raw images. For each animal, 3–5 random, non-overlapping fields of view were selected for analysis. The Median Fluorescence Intensity (MFI) within the ROIs was measured. Background fluorescence was determined by measuring three random signal-free areas outside the tissue on the same section and averaged. The final data was expressed as background-corrected MFI in arbitrary units (a.u.). The mean MFI of the 3–5 technical replicates per mouse were calculated to yield a single biological value.

VSMCs were stained with anti-SPOCK2 (Santa Cruz Technology, Cat# sc-515691) and anti-MMP2 (Proteintech, Cat# 10373-2-AP). Fluorescence images were captured using Leica confocal microscope SP8. To evaluate signal intensity and co-localization, high-power field images (n = 5 randomly selected fields per sample) were analyzed using ImageJ software (NIH, Bethesda, USA). All assessments were performed by investigators in a blinded manner.

### Isolation and culture of mouse primary bone marrow-derived macrophages and mouse primary aortic smooth muscle cells

Eight-week-old C57BL/6 mice were euthanized via isoflurane inhalation. Bone marrow from the femur and tibia was flushed out with culture medium (DMEM supplemented with 10% FBS), followed by centrifugation. The resuspended bone marrow cells were filtered through a 70-μm cell strainer and then placed in a macrophage culture medium (DMEM supplemented with 10% FBS and 20ng/mL M-CSF). Bone marrow cells were cultured at 37 °C in a 5% CO_2_ incubator, and the medium was refreshed every three days. After seven days of culture, the resulting bone marrow-derived macrophages (BMDMs) were used for subsequent experiments.

Aortas were obtained from eight-week-old C57BL/6 mice, and aortas from three mice were pooled for one biological replicate. Mice were euthanized via isoflurane inhalation, and the entire aorta, extending to the renal bifurcation, was carefully exposed. To remove residual blood, the aorta was perfused using a tuberculin syringe containing sterile PBS. Under a dissecting microscope, surrounding adipose and adventitial tissues were meticulously removed. The aorta was dissected out and transferred to culture medium. Next, the vessel was cut into 1- to 2-mm pieces and incubated at 37 °C in a digestion solution composed of 2 mg/mL collagenase type I (Worthington Biochemical Corporation, NJ, USA) and 1mg/mL dispase II (Sigma-Aldrich, MO, USA) for one hour. After removing the digestion solution, the aortic fragments were transferred to complete DMEM medium and cultured undisturbed at 37 °C with 5% CO_2_ for five days. The purity of VSMCs was assessed by α-SMA (Cell Signaling Technology, Cat# 19245) immunostaining. All experiments were performed using VSMCs from passages 2 to 5.

### Human coronary artery smooth muscle cells

Human coronary artery smooth muscle cells (hCASMCs) and the corresponding culture medium were obtained from Procell (Wuhan, China). The cells were treated with (10% v/v) serum from healthy controls and KD patients for 24 hours. Cells at passage 2 to 5 were utilized for the experiments.

### Transwell co-culture system

BMDMs were seeded onto Transwell inserts with permeable membranes (pore size: 0.45 μm) and placed over a 6-well plate pre-seeded with VSMCs. To induce macrophage-mediated proinflammatory paracrine effects, CAWS (20 ng/mL) was directly added to the upper chamber. Following a 24-hour incubation period, VSMCs in the lower chamber were collected for subsequent experiments.

### Cell transduction and transfection

The lentivirus construct for SPOCK2 was obtained from OBIO Technology (Shanghai, China). Briefly, VSMCs were transfected with either SPOCK2 overexpression lentivirus (pcSlenti-EF1-EGFP-P2A-Puro-CMV-SPOCK2-3xFLAG-WPRE, Lenti-SPOCK2) and control lentivirus vectors (pcSlenti-EF1-EGFP-P2A-Puro-CMV-MCS-3xFLAG-WPRE, Lenti-Vector) at an appropriate multiplicity of infection (MOI). Stably transduced cells were selected using puromycin treatment over a period of one week.

For transient expression experiments in HEK293T cells, cells were co-transfected with HA-tagged SPOCK2 and Flag-tagged MMP2 plasmids (or respective control vectors) using Lipofectamine 3000 (Thermo Fisher Scientific, USA) according to the manufacturer’s protocol. Culture media were refreshed 6 hours post-transfection, and cells were harvested 48 hours post-transfection for co-immunoprecipitation (Co-IP) and downstream molecular analyses.

### Gene expression datasets analysis

Transcriptional profiles of peripheral blood samples from patients with KD and their corresponding healthy controls were obtained from the Gene Expression Omnibus (GEO) datasets (GSE68004, GSE73461, and GSE18606). Specifically, data were collected from 37 healthy individuals and 74 patients with complete KD in GSE68004; data were collected from 55 healthy individuals and 78 patients with KD in GSE73461; data were collected from 9 healthy individuals and 8 IVIG-resistant KD patients at the acute phase in GSE18606. Data analysis was performed by GEO2R to identify differentially expressed genes (DEGs). DEGs were defined based on *p*-value < 0.05 and |log_2_FC| >1. A subset of genes encoding members of the SPARC protein family were selected for further analysis, including *SPARC*, *SPARCL1*, *SPOCK1*, *SPOCK2*, *SPOCK3*, *SMOC1*, *SMOC2*, and *FSTL1*. Commonly expressed SPARC family genes across datasets GSE68004, GSE73461, and GSE18606 were then identified.

Public RNA sequencing datasets (GSE141072) were retrieved from GEO database. Data were collected from 5 male mice from PBS group and 5 male mice from LCWE-treated group. Differential expression analysis between control and LCWE-treated groups was performed using the R package DESeq2 (v1.42.0). Raw gene counts were normalized using DESeq2’s median-of-ratios method, and dispersion parameters were estimated to model gene-wise variance. DEGs for GSE141072 were defined based on adjusted *p*-value < 0.05 and |log_2_FC| >2, derived using the Benjamini-Hochberg false discovery rate (FDR) correction method. All computational workflows, statistical testing, and figure renderings were conducted within the R software environment (version 4.5.1) using packages including DESeq2, ggplot2, and pheatmap.

### RNA sequencing and data analysis

Total RNA was extracted from the mouse abdominal aorta using TRIzol™ Reagent (Invitrogen, MA, USA) according to the manufacturer’s instructions. RNA purity and concentration were evaluated by NanoDrop 2000 spectrophotometer (Thermo Fisher Scientific, MA, USA). RNA integrity was assessed using Agilent 2100 Bioanalyzer (Agilent Technologies, CA, USA). The libraries were constructed using the VAHTS Universal V6 RNA-seq library Prep Kit for Illumina (Vazyme, Nanjing, China) according to the manufacturer’s instructions. The transcriptome sequencing and analysis were conducted by OE Biotech, Inc. (Shanghai, China).

The libraries were sequenced on an Illumina NovaSeq 6000 Sequencing System (Illumina, CA, USA) yielding 150bp paired end reads. About 47M raw reads for each sample were generated. Raw reads in FASTQ format were first processed using FastQ, and low-quality reads were removed to obtain clean reads. Approximately 46 million clean reads for each sample were retained for subsequent analyses. The clean reads were mapped to the reference genome using HISAT2. FPKM of each gene was calculated, and the read counts of each gene were obtained by HTseq-count. PCA analysis was performed using R (version 3.2.0) to evaluate the biological duplication of samples. Differential expression analysis was performed using DESeq2. Q value < 0.05 and foldchange > 1 were set as the threshold for significantly differentially expressed genes (DEGs). GSEA analysis was performed to screen gene distribution. Bioinformatic analysis was performed using the OECloud tools at https://cloud.oebiotech.com/task/. All primary data can be accessed at China National Center of Bioinformation CRA029797.

### qRT-PCR

Complementary DNA was synthesized from 1000 ng of total RNA using a HiScriptIII RT SuperMix kit (Vazyme, Nanjing, China). Real-time quantitative PCR (qPCR) was done using ChamQ SYBR qPCR MasterMix kit (Vazyme, Nanjing, Jiangsu, China). Each sample was performed in duplicate or triplicate in each experiment. The 2^-ΔΔCT^ method was used for data analysis, with *Gapdh* as the endogenous control. Primers used for qPCR are listed in **Supplementary Table 1**.

### ELISA

Serum obtained from mouse peripheral blood was stored at -80 °C until assay. The levels of serum IL-1β, IL-6, and TNF-α were measured using ELISA Kits (RayBiotech, GA, USA) following the manufacturer’s instructions. All samples were measured on a single 96-well plate for each cytokine.

### Western immunoblotting

Frozen tissues and cultured cells were lysed in RIPA lysis buffer (Sigma-Aldrich, MO, USA) with protease and phosphatase cocktail inhibitors (Roche, NV, USA) to yield total protein. Protein concentration was determined using the BCA protein assay kit (Thermo Fisher Scientific, MA, USA). Proteins were separated using 8% - 12% SDS-polyacrylamide gel electrophoresis and transferred to PVDF membranes (Millipore, MO, USA). After blocking with 5% skim milk, membranes were hybridized with anti-NLRP3 (Cell Signaling Technology, Cat# 15101), anti-Cleaved Caspase-1(Cell Signaling Technology, Cat# 89332), anti-Cleaved IL-1β (Cell Signaling Technology, Cat# 63124), anti-α-SMA (Cell Signaling Technology, Cat# 19245), anti-Tagln (Cell Signaling Technology, Cat# 40471), anti-PCNA (Cell Signaling Technology, Cat# 13110), anti-periostin (Proteintech, Cat# 66491-1-Ig), anti-vimentin (Proteintech, Cat# 60330-1-Ig), anti-collagen type I (Proteintech, Cat# 67288-1-Ig), anti-collagen type III (Proteintech, Cat# 22734-1-AP), and anti-GAPDH (Proteintech, Cat# 60004-1-Ig), anti-MMP2 (ABclonal, Cat# A19080), anti-SPOCK2 (Santa Cruz Biotechnology, Cat# sc-515681), and anti-Flag (Sigma-Aldrich, Cat# B3111) at 4 °C overnight. Subsequently, anti-mouse IRDye (LICORbio™, Cat# 926-32220) or anti-rabbit IRDye secondary antibodies (LICORbio™, Cat# 926-32213) were incubated at room temperature for 1 hour with the appropriate primary antibody host. The signals were produced using a LICOR Odyssey Classic Imager (LICORbio™, NE, USA) and analyzed using LICOR Image Studio Software (version 6.0). If necessary, the membranes were stripped using Restore™ western blot stripping buffer (Thermo Fisher Scientific, MA, USA), followed by additional blotting.

### Co-immunoprecipitation

Cells were lysed with Pierce™ IP lysis buffer (Thermo Fisher Scientific, MA, USA), and cell debris was removed by centrifugation. Resulting cell lysates were incubated with Pierce™ Anti-DYKDDDDK Magnetic beads and Anti-HA Magnetic beads (Thermo Fisher Scientific, MA, USA) following the manufacturer’s instructions. After a series of washing steps, captured proteins were eluted by boiling with 4x Laemmli sample buffer containing DTT (Bio-Rad Laboratories, CA, USA) and then proceeded to immunoblotting.

### Gel zymography for analysis of MMP2

Gel zymography assay was performed in accordance with the manuscript’s protocol (Real-Times Biotechnology Co., Beijing, China). Briefly, VSMCs lysates were mixed with non-reducing sample buffer and loaded into the wells of a 7.5% acrylamide gel containing gelatin. Electrophoresis was performed at 150V until the bands were well separated. The gel was then subjected to vigorous washing with washing buffer, followed by incubation in incubation buffer and staining solution. Subsequently, destaining was carried out using the destaining buffer. The resulting bands appeared as white bands against a blue background.

### Molecular docking analysis

To investigate the protein interaction between SPOCK2 and MMP2, we performed in silico molecular docking analysis. The three-dimensional (3D) structures of human SPOCK2 and MMP2 were retrieved from the UniProt database. Protein-protein docking was conducted using the HADDOCK (High Ambiguity Driven protein-protein DOCKing) web server to predict the binding affinity and orientation of the SPOCK2-MMP2 complex. The docking results were further processed using the PDBePISA (Proteins, Interfaces, Structures, and Assemblies) server to identify the specific amino acid residues involved in the interaction interface and to calculate the binding free energy. Finally, the binding interface and 3D docking models were visualized and rendered using PyMOL software (Schrödinger, LLC) to illustrate the spatial configuration of the protein complex.

### Statistics

Data are expressed as means ± SEM. For comparison between 2 unpaired groups, a two-tailed Student’s *t*-test was performed for all normally distributed data. The Mann-Whitney *U* test was used for data that did not have a normal distribution. For comparisons across multiple groups, one-way analysis of variance (ANOVA) followed by Tukey’s *post hoc* test was conducted for normally distributed data. For multiple group comparisons of non-normally distributed data, the Kruskal-Wallis test followed by Dunn’s *post hoc* test was used. *p* < 0.05 was considered statistically significant. All statistical analyses were performed using the GraphPad Prism 9 software.

## Results

### VSMCs exhibited a fibroblast-like phenotype in the aortic root of CAWS-induced murine model of KD vasculitis

To investigate whether a phenotype switch of VSMC is involved in the pathogenesis of cardiovascular lesions during KD, we used CAWS to induce KD vasculitis in a murine model (**Figure 1A**). As shown in **Figure 1B**, H&E staining revealed a significant increase in immune cell infiltration in the aortic root region of the CAWS-injected mice. Furthermore, Masson’s trichrome staining demonstrated a significant increase in interstitial and perivascular fibrosis in the aortic root of the CAWS group compared with the PBS (Control) group (**Figure 1C**). Quantitative analysis of heart vessel inflammation scores in the aortic root further confirmed that CAWS injection induced significant myocardial inflammation (**Figure 1D**). Moreover, the relative mRNA expression levels of proinflammatory cytokines, including *Il1b, Il6*, and *Tnfa*, were significantly upregulated in the aortic root of the CAWS-injected mice (**Figure 1E**). Given the well-established role of the NLRP3 inflammasome in the pathogenesis of KD, we further assessed the activation status of the NLRP3 inflammasome in the aortic root following CAWS injection. Western blot analysis showed significantly increased protein levels of NLRP3, cleaved caspase-1, and cleaved IL-1β in the aortic root of the CAWS group compared with controls (**Figures 1F, G**). Collectively, these findings indicate that CAWS injection effectively induces vasculitis in this mouse model.

**Figure 1:**
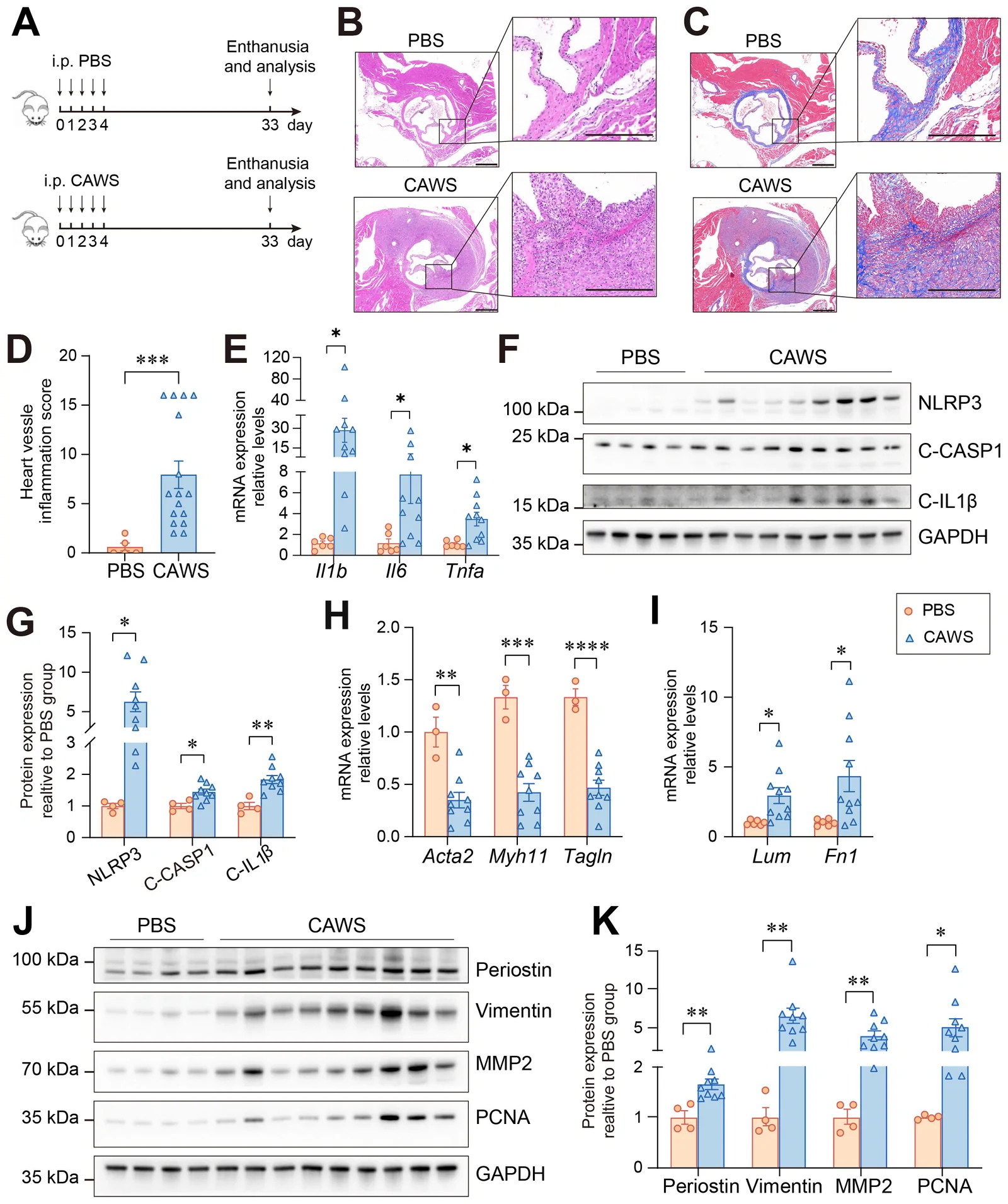
Phenotypic switching of VSMCs in the aortic root of a murine model of CAWS-induced vasculitis. **(A)** Schematic diagram illustrating the experimental protocol. **(B)** Representative histological images showing H&E-stained and **(C)** Masson’s trichrome-stained transverse sections of aortic root from PBS- and CAWS-treated mice 28 days after the last injection. Scale bars: 0.5 mm. **(D)** Quantitative analysis of heart vessel inflammation scores in the aortic root of PBS-treated (n=5) and CAWS-treated (n=16) mice. **(E)** Relative mRNA expression levels of *Il1b*, *Il6*, and *Tnfa* in the aortic root of PBS-treated (n=6) and CAWS-treated (n=10) mice. **(F)** Representative Western immunoblots and **(G)** quantitative densitometric estimation of NLRP3, cleaved caspase-1 (C-CASP1), and cleaved IL-1β (C-IL1β) in the aortic root in PBS-treated (n=4) and CAWS-treated (n=9) mice. **(H)** Relative mRNA expression levels of *Acta2*, *Myh11*, and *Tagln* **(H)** in the aortic root of PBS-treated (n=3) and CAWS-treated (n=9) mice. **(I)** Relative mRNA expression levels of *Lum* and *Fn1* **(I)** in the aortic root of PBS-treated (n=6) and CAWS-treated (n=10) mice. **(J)** Representative Western immunoblots and **(K)** densitometric quantification of periostin, vimentin, MMP2, and PCNA in the aortic root of PBS-injected (n=4) and CAWS-injected (n=9) mice. Data are presented as mean ± SEM. *p < 0.05, **p < 0.01, ***p < 0.001, **** *p*<0.0001.

We further examined the mRNA expression levels of VSMC contractile markers, including *Acta2*, *Myh11*, and *Tagln*, which were significantly decreased in the aortic root of CAWS-injected mice compared with controls (**Figure 1H**). Moreover, as luminal myofibroblast proliferation (LMP) represents a predominant pathological feature during the progression of KD vasculitis, our findings demonstrated that the mRNA expression levels of fibroblast-related markers, such as *Lum* and *Fn1*, were significantly increased in the aortic root of CAWS-treated mice (**Figure 1I**). Additionally, the protein levels of periostin, vimentin, MMP2, and PCNA were significantly increased in the CAWS group (**Figures 1J, K**). Taken together, these results suggest that VSMCs adopt a synthetic phenotype and exhibit the potential to transition to fibroblast-like VSMCs during the pathophysiological process of KD.

### A pathogenic phenotype switch of VSMCs drove CAWS-induced abdominal aortic aneurysms

Aortic aneurysm is a severe complication of KD, which can lead to thrombosis, rupture, and even sudden death. In the CAWS-induced mouse model of vasculitis, we observed that CAWS administration led to significant development of abdominal aortic aneurysm and elastin degradation in the vascular wall (**Figures 2A–E**). Moreover, the mRNA levels of proinflammatory cytokines, including *Il1b*, *Il6* and *Tnfa*, as well as *Clec4n* and *Syk* were significantly elevated (**Figure 2F**). In contrast, the expression of VSMC contractile phenotype-related markers, such as *Acta2, Tagln*, and *Myh11*, was significantly reduced in the abdominal aorta of CAWS-injected mice (**Figure 2G**). Consistently, the protein levels of α-SMA and Tagln were significantly decreased in the abdominal aorta of CAWS-treated mice (**Figures 2H, I**), aligning with their corresponding gene expression profiles. Conversely, the protein levels of PCNA, periostin, vimentin, and MMP2 were notably increased in the abdominal aorta of CAWS-treated mice (**Figures 2H–K**). These findings suggest that the acquisition of a fibroblast-like phenotype switch by VSMCs contributes to the pathogenesis of CAWS-induced aortic aneurysm.

**Figure 2:**
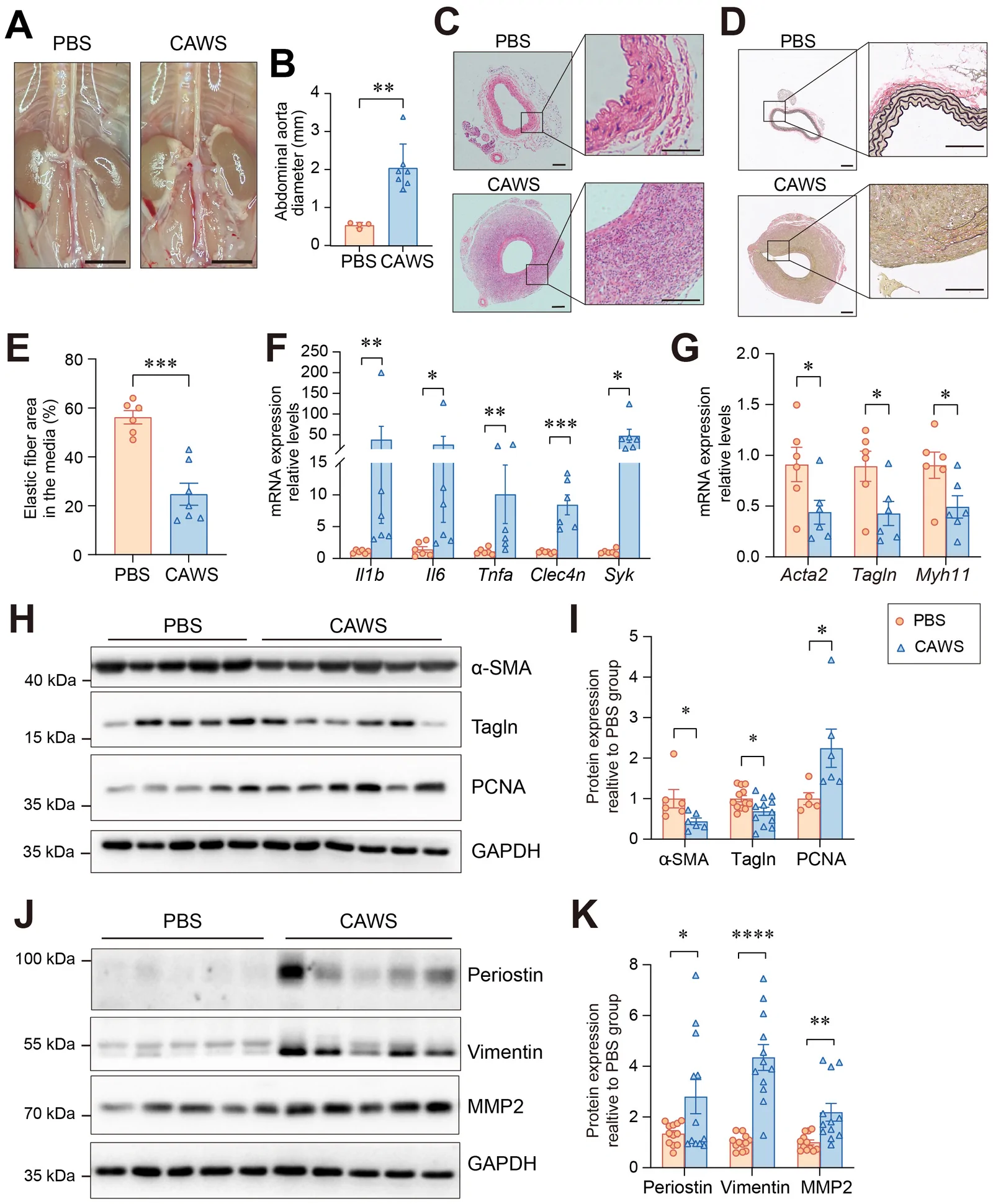
Phenotypic switching of VSMCs in CAWS-induced abdominal aortic aneurysm. **(A)** Representative gross images of abdominal aorta. Scale bars: 5mm. **(B)** Quantitative measurement of the abdominal aorta diameter in PBS-treated (n=4) and CAWS-treated (n=7) mice. **(C–D)** Representative transverse histological sections stained with H&E **(C)** and EVG **(D)** from PBS-treated and CAWS-treated mice. Scale bars: 0.1mm. **(E)** Quantitative measurement of elastic fiber within the vessel wall in PBS-treated (n=6) and CAWS-treated (n=7) mice. **(F–G)** Relative mRNA expression levels of *Il1b, Il6, Tnfα, Clec4n, and Syk* **(F)**, and *Acta2*, *Tagln*, and *Myh11* **(G)** in the abdominal aorta of PBS-treated and CAWS-treated mice (n=6 per group). **(H)** Representative Western immunoblots and **(I)** quantitative densitometric estimation of α-SMA, Tagln, and PCNA protein levels in the abdominal aorta. α-SMA and PCNA (n=6 per group), Tagln (n=12 per group). **(J)** Representative Western immunoblots and **(K)** corresponding densitometric quantification of periostin, vimentin, and MMP2 protein levels in the abdominal aorta (n=12 per group). Data are presented as mean ± SEM. *p < 0.05, **p < 0.01, ***p < 0.001, ****p < 0.0001.

### SPOCK2 was downregulated in Kawasaki disease

We analyzed the transcriptional profiles of peripheral blood from patients with KD using the GEO datasets GSE68004, GSE73461, and GSE18606. Following gene screening for SPARC family members (*FSTL1, SMOC1, SMOC2, SPOCK1, SPOCK2, SPOCK3, SPARC, SPARCL1*) among differentially expressed genes (DEGs) identified in each dataset, we found that *SPOCK2* was significantly downregulated in KD patients during the acute phase compared with healthy controls (**Supplementary Figures 1A–C**). Notably, *SPOCK2* was the only gene within the SPARC family that showed consistently reduced expression across all datasets in KD patients (**Figure 3A**). Consistently, we examined that the mRNA expression levels of *SPOCK2* were significantly downregulated in the peripheral blood of KD patients compared to healthy controls (**Figure 3B**).

**Figure 3:**
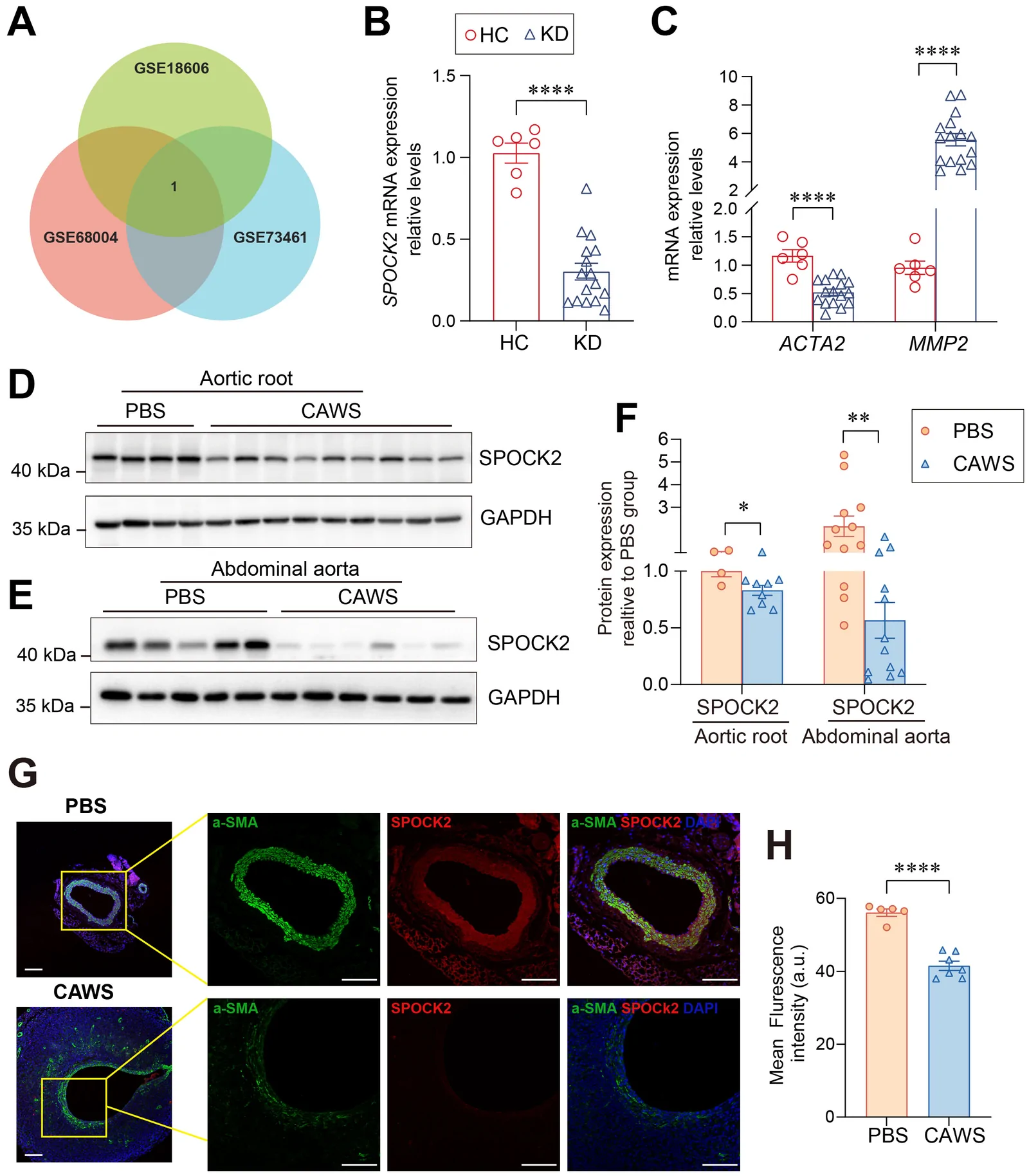
Identification of SPOCK2 in patients with Kawasaki disease and in a murine model of CAWS-induced vasculitis. **(A)** Venn diagram showing the overlapping SPARC family gene across the GSE18606, GSE73461, and GSE68004 datasets. **(B)** Relative mRNA expression levels of *SPOCK2* in human peripheral blood samples from healthy controls (HC, n=6) or KD patients (n=16). **(C)** Relative mRNA expression levels of *ACTA2* and *MMP2* in hCASMCs exposed to serum from healthy controls (n=6) or KD patients (n=16). **(D–E)** Representative Western immunoblots of SPOCK2 in the aortic root **(D)** (PBS, n=4; CAWS, n=9) and abdominal aorta **(E)** (n=12 per group) of PBS-treated and CAWS-injected mice, with **(F)** corresponding quantitative densitometric analysis. **(G)** Representative immunofluorescent images of transverse sections of the abdominal aorta from PBS- and CAWS-injected mice. α-SMA was stained in green, SPOCK2 in red, and nuclei were counterstained with DAPI (blue). Scale bars: 100 μm. **(H)** Quantitative analysis of SPOCK2 fluorescence signal intensity in the abdominal aortic wall measured using Image J software. Signal intensity is expressed as Mean Fluorescence Intensity in arbitrary units (a.u.) (PBS, n=5 mice; CAWS, n=7 mice). Individual data points represent independent biological replicates. Data are represented as mean ± SEM. *p < 0.05, **p < 0.01, ****p < 0.0001.

To explore the potential role of SPOCK2 in VSMCs during KD, we established ex vivo conditions resembling KD. Human coronary artery smooth muscle cells (hCASMCs) were exposed to serum obtained from KD patients during the acute phase prior to intravenous immunoglobulin (IVIG) treatment. mRNA levels of *ACTA2* were reduced considerably (**Figure 3C**), a finding further confirmed at the protein level (**Supplementary Figures 2A, B**). In contrast, *MMP2* mRNA expression was significantly upregulated (**Figure 3C**), and the protein expression of the proliferation marker, PCNA, was significantly increased in hCASMCs after KD serum stimulation (**Supplementary Figures 2A, C**). Importantly, the protein expression levels of SPOCK2 were significantly decreased in both aortic root and abdominal aorta of CAWS-treated mice (**Figures 3D–F**). Furthermore, Immunofluorescence staining demonstrated a significant reduction in SPOCK2 expression in the abdominal aorta following CAWS stimulation, along with VSMCs migration into the intima of the abdominal aorta (**Figures 3G, H**).

To confirm that SPOCK2 expression is not restricted to fugal pattern-recognition pathways activated by CAWS, we evaluated mRNA expression levels of typical signaling pathway induced by LCWE, such as *Tlr2* and *Myd88*, as well as *Spock2* in a public transcriptomic dataset from the abdominal aorta of LCWE-induced murine vasculitis model (GSE141072). Transcriptomic profile revealed that mRNA expression levels of *Tlr2* and *Myd88* were significantly increased in abdominal aorta of LCWE-stimulated mice compared to controls (**Supplementary Figures 3A–D**). Moreover, consistent with our CAWS findings, *Spock2* mRNA levels were significantly decreased in abdominal aorta of LCWE-stimulated mice compared to controls (**Supplementary Figure 3E**). Therefore, SPOCK2 depletion is a convergent hallmark across distinct KD vasculitis models.

### Overexpression of SPOCK2 mitigated CAWS-induced vasculitis and systemic inflammation

To investigate the role of SPOCK2 in CAWS-induced vasculitis, we constructed an AAV2/9 vector encoding the *Spock2* gene sequence (AAV2/9-SPOCK2) to induce SPOCK2 overexpression in the myocardium specifically. Mice were administered AAV2/9-SPOCK2 and control vector via a single tail vein injection one week prior to CAWS injection (**Figure 4A**). Twenty-eight days after last CAWS injection, AAV2/9-mediated SPOCK2 overexpression significantly attenuated immune cell infiltration and fibrosis in the aortic root region (**Figures 4B–D**). Moreover, serum levels of IL-1β, IL-6, and TNF-α were significantly lower in the AAV2/9-SPOCK2-injected mice compared with the control mice (**Figures 4E–G**). Additionally, the protein expression level of SPOCK2 was significantly increased in the AAV2/9-SPOCK2 group compared to the control group, resulting in an effective inhibition of MMP2 protein expression after CAWS induction (**Figures 4H, I**).

**Figure 4:**
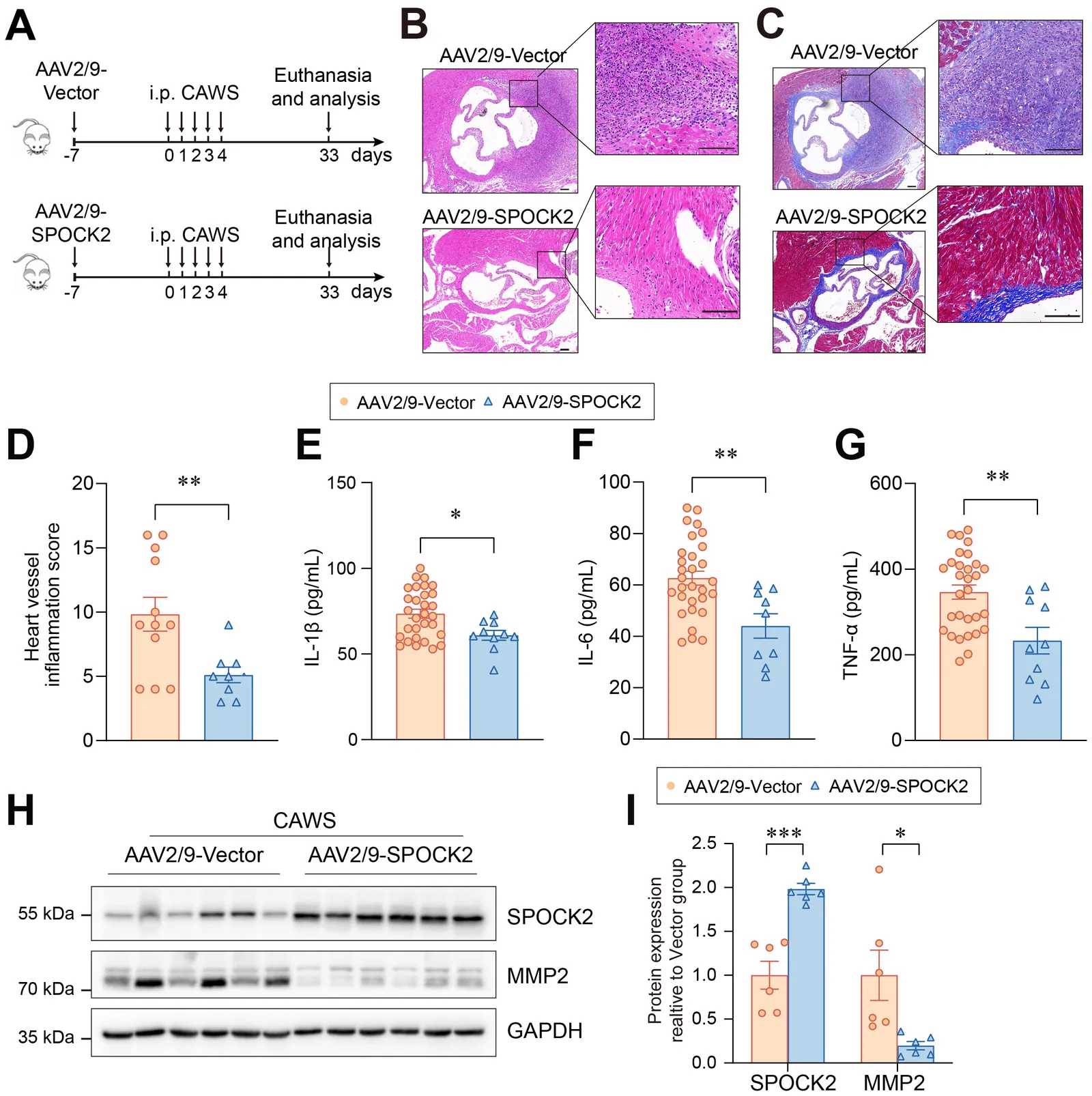
Overexpression of SPOCK2 attenuated CAWS-induced vasculitis in mice. **(A)** Schematic diagram of the experimental protocol. **(B–C)** Representative images showing H&E-stained **(B)** and Masson’s trichrome-stained **(C)** transverse sections of aortic root in AAV2/9-Vector and AAV2/9-SPOCK2-treated mice at 28 days post-CAWS injection. Scale bars: 0.1 mm. **(D)** Quantitative data of heart vessel inflammation scores in AAV2/9-Vector (n=12) and AAV2/9-SPOCK2 (n=9) groups. **(E–G)** Serum levels of IL-1β **(E)**, IL-6 **(F)**, and TNF-α **(G)** measured by ELISA in AAV2/9-Vector (n=30) and AAV2/9-SPOCK2 (n=10) groups. **(H–I)** Representative Western immunoblots **(H)** and **(I)** corresponding densitometric quantification of SPOCK2 and MMP2 expression in the aortic root (n=6 per group). Data are presented as mean ± SEM. *p < 0.05; **p < 0.01; ***p < 0.001.

### SPOCK2 overexpression prevented abdominal aortic wall remodeling by restoring VSMC contractile homeostasis

To further investigate the role of SPOCK2 in modulating CAWS-induced abdominal aortic aneurysm formation, we initially assessed the protein expression levels of SPOCK2 in abdominal aorta following CAWS induction. The results demonstrated a significant upregulation of SPOCK2 protein levels in the abdominal aorta of mice treated with AAV2/9-SPOCK2 compared to those receiving the empty vectors (**Figures 5A, B**). Moreover, the diameter of the abdominal aorta was significantly decreased in the AAV2/9-SPOCK2 group (**Figures 5C, D**). Collectively, these findings indicate that AAV2/9-mediated overexpression of SPOCK2 effectively attenuates CAWS-induced abdominal aortic aneurysm formation.

**Figure 5:**
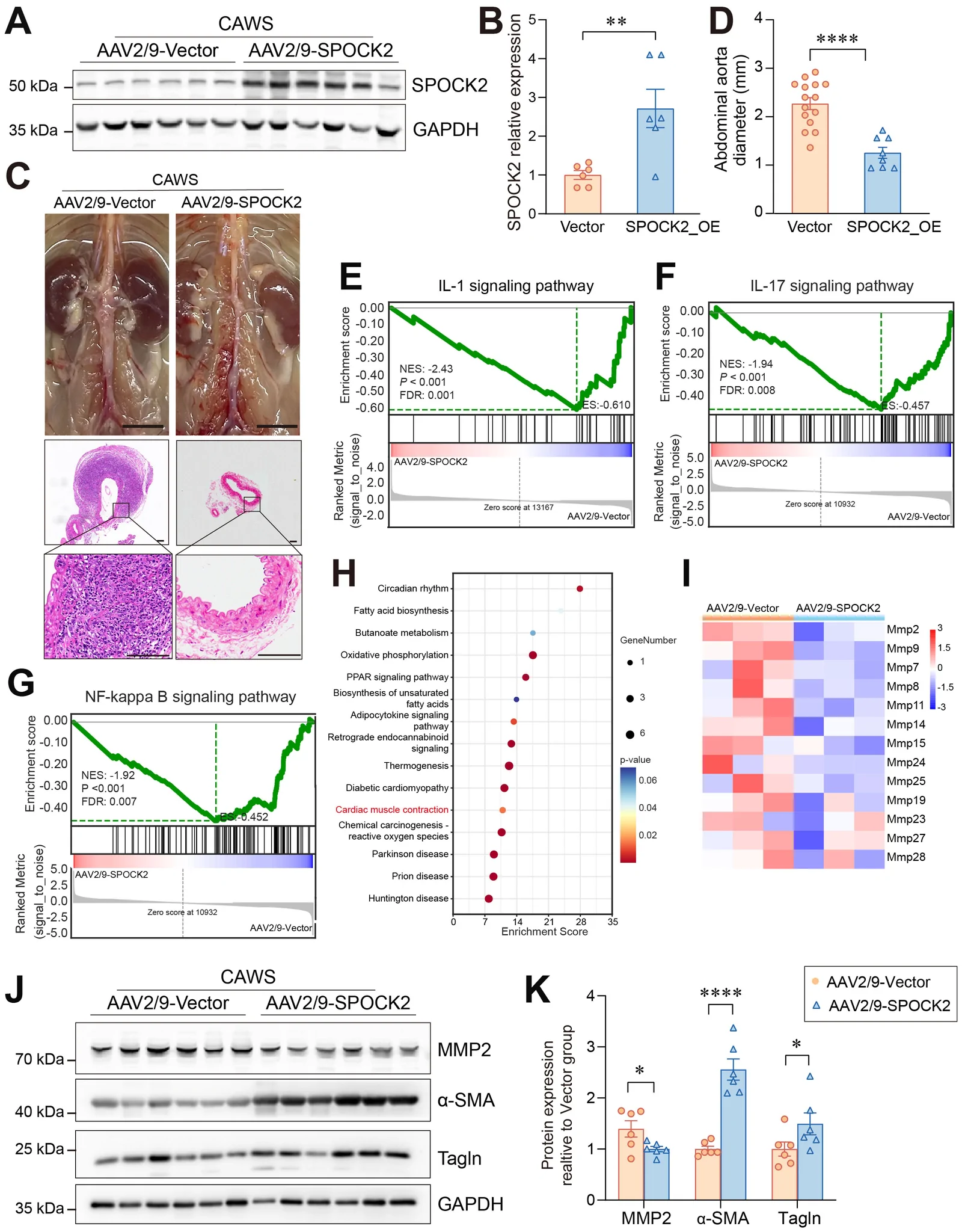
Overexpression of SPOCK2 attenuated CAWS-induced abdominal aortic aneurysm formation. **(A)** Representative Western immunoblots **(A, B)** quantitative densitometric estimation of SPOCK2 protein levels in the abdominal artery in AAV2/9-Vector- and AAV2/9-SPOCK2 (SPOCK2_OE)-injected mice (n=6 per group). **(C)** Representative gross morphological images (upper panels; scale bars: 5 mm) and transverse histological sections stained with H&E (lower panels; scale bars: 100 μm) of the abdominal aorta and **(D)** quantitative measurements of abdominal aorta diameter 28 days after the final CAWS injection (AAV2/9-Vector, n=15; AAV2/9-SPOCK2, n=8). **(E–G)** GSEA scoring curves depicting enrichment profiles for IL-1 **(E)**, IL-17 **(F)**, and NF-κB **(G)** signaling pathways in the abdominal aorta (n=3). **(H)** KEGG enrichment analysis of DEGs between the AAV2/9-SPOCK2 group and AAV2/9-vector group, displaying the top 15 upregulated pathways. **(I)** Heatmap illustrated differential expression profiles of MMPs in the abdominal aorta (n=3 per group). **(J)** Representative Western immunoblots and **(K)** quantitative densitometric analysis of MMP2, α-SMA, and Tagln protein levels in the abdominal aorta (n=6 per group). Data are represented as mean ± SEM. *p < 0.05, **p < 0.01, ****p < 0.0001.

To elucidate the underlying mechanisms of SPOCK2 in CAWS-induced abdominal aortic wall remodeling, we performed RNA sequencing to analyze gene expression changes in the abdominal aorta of AAV2/9-SPOCK2-injected mice and control mice following CAWS induction. The principal component analysis (PCA) plot and heatmap showed a clear distinction between the AAV2/9-SPOCK2 group and the control group (**Supplementary Figures 4A, B**). Gene set enrichment analysis (GSEA) revealed that genes associated with cytokine-mediated signaling pathways, including IL-1, IL-17, and NF-κB signaling pathways, were significantly enriched in the control group compared with AAV2/9-SPOCK2 treatment group following CAWS stimulation (**Figures 5E–G**). Moreover, overexpression of SPOCK2 significantly attenuated CAWS-induced *Clec4n* and *Syk* upregulation (**Supplementary Figures 4C, D**). Differentially expressed genes (DEGs) were visualized in a volcano plot (**Supplementary Figure 4E**). Kyoto encyclopedia of genes and genomes (KEGG) enrichment analysis indicated that DEGs related to cardiac muscle contraction were significantly upregulated in the AAV2/9-SPOCK2 group compared to the control group (**Figure 5H**). Consistently, the Gene Ontology (GO) biological process (BP) annotations revealed that genes significantly upregulated by SPOCK2 overexpression were heavily enriched in structural muscle maintenance and development, specifically featuring skeletal muscle thin filament assembly and cardiac muscle cell development (**Supplementary Figure 4F**, Upper Panel). This structural rescue was further reinforced by highly significant enrichments in cellular component (CC) and molecular function (MF) terms, including the muscle myosin complex, sarcoplasmic reticulum, Z disc, I band, and the structural constituent of muscle (**Supplementary Figure 4F**, middle panel and lower panel). Together, these congruent transcriptomic profiles demonstrate that SPOCK2 overexpression actively stabilizes the core contractile apparatus under pathologically stressful conditions.

Given that ECM degradation and remodeling are critical downstream pathological events driven by the inflammatory cascades during aneurysm formation, we subsequently focused on the expression profiles of ECM remodeling enzymes. Moreover, a heatmap of gene expression profiles of Matrix metalloproteinases (MMPs) showed that MMP2 expression was specifically downregulated in the abdominal aorta of AAV2/9-SPOCK2-injected mice after CAWS induction compared to controls (**Figure 5I**). Consistently, the protein expression level of MMP2 was significantly reduced in the abdominal aorta of AAV2/9-SPOCK2 group (**Figures 5J, K**). In contrast, VSMC-related markers, α-SMA and Tagln, were significantly elevated in the abdominal aorta of AAV2/9-SPOCK2 group compared to the control group (**Figures 5J, K**). In conclusion, these findings suggest that overexpression of SPOCK2 substantially rescues CAWS-induced abdominal aortic aneurysm and restores the contractile phenotype of VSMCs.

### SPOCK2 protected against the inflammation-driven VSMC phenotype switch

To investigate whether SPOCK2 is involved in the phenotype switch of VSMCs during KD vasculitis, we utilized a transwell co-culture system to simulate the inflammatory environments of KD vasculitis *in vitro* (**Figure 6A**). Briefly, bone marrow-derived macrophages (BMDMs) were cultured in transwell permeable inserts and pretreated with CAWS, then the inserts were transferred to a culture system containing VSMCs. Our results revealed a significant upregulation in mRNA levels of pro-inflammatory cytokines, including *Il1*, *Il6*, and *Nos2*, in CAWS-induced VSMCs compared with control cells (**Figure 6B**). Moreover, the mRNA expression levels of ECM remodeling enzymes, such as *Mmp2* and *Mmp9*, were significantly increased in the CAWS-induced VSMCs (**Figure 6C**). In contrast, VSMCs-specific markers, such as *Acta2*, *Tagln*, and *Myh11*, and *Spock2* were significantly downregulated in the CAWS-induced VSMCs (**Figure 6D**). These mRNA-level observations were corroborated at the protein expression level (**Figures 6E, F**). In addition, the protein expression levels of periostin, collagen type Iα, and collagen type IIIα were significantly increased in the CAWS-induced VSMCs (**Figures 6E, F**).

**Figure 6:**
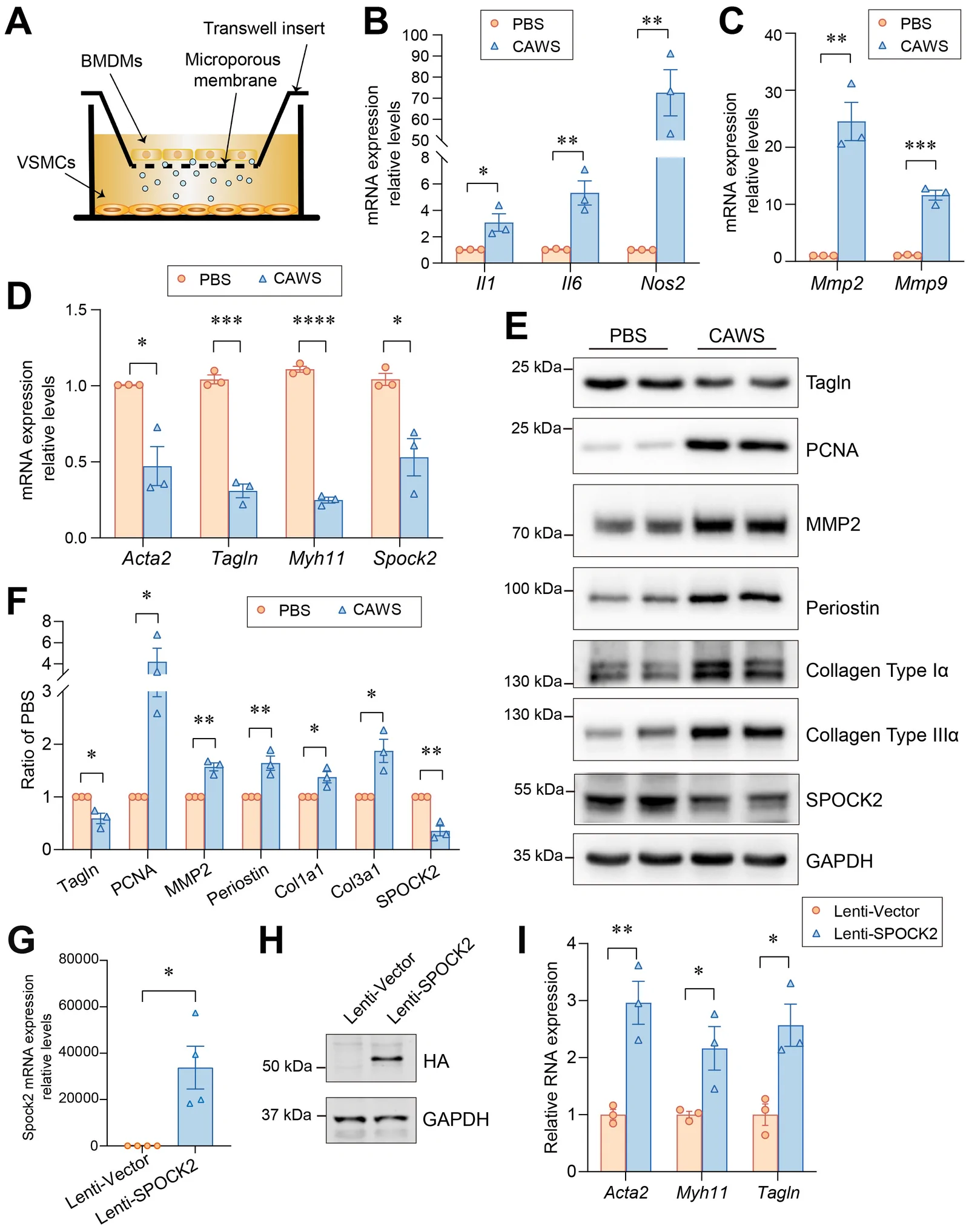
Overexpression of SPOCK2 attenuated CAWS-induced VSMC phenotypic switching *in vitro*. **(A)** Schematic diagram of the transwell co-culture system. **(B–D)** Relative mRNA expression levels of *Il1b*, *Il6*, and *Nos2* **(B)**; *Mmp2* and *Mmp9* **(C)**; and *Acta2*, *Tagln*, *Myh11*, and *Spock2* **(D)** in PBS- and CAWS-treated VSMCs (n=3 per group). **(E)** Representative Western immunoblots and **(F)** densitometric quantification of Tagln, PCNA, MMP2, periostin, collagen type Iα, collagen type IIIα, and SPOCK2 protein levels (n=3 per group). **(G)** Relative mRNA levels of *Spock2* in VSMCs transfected with HA-tagged Lenti-SPOCK2 (n=4 per group). **(H)** Representative Western immunoblots of HA-tag expression (n=3 per group). **(I)** Relative mRNA levels of *Acta2*, *Myh11*, and *Tagln* in Lenti-Vector- and Lenti-SPOCK2-transfected VSMCs following CAWS induction (n=3 per group). Data are represented as mean ± SEM. *p < 0.05, **p < 0.01, ***p < 0.001, ****p < 0.0001.

Furthermore, we generated lentiviral vector-mediated SPOCK2-overexpressing (Lenti-SPOCK2) VSMCs. The mRNA and protein levels of SPOCK2 were significantly increased in Lenti-SPOCK2 VSMCs compared with control cells (**Figures 6G, H**). The inhibitory effects of CAWS on the contractile markers of VSMCs, including *Acta2*, M*yh11*, and *Tagln*, were significantly attenuated by SPOCK2 overexpression (**Figure 6I**). These data indicate that VSMCs undergo a phenotypic transition toward a synthetic state characterized by enhanced proliferation and extensive ECM synthesis during the development of CAWS-induced vasculitis, which can be blunted by SPOCK2.

### SPOCK2 physically associated with MMP2 and attenuated its activation

To elucidate the underlying mechanisms of SPOCK2 in VSMC phenotype switch during KD, SPOCK2 was overexpressed via lentivirus transduction in VSMCs. Both empty vector- and Lenti-SPOCK2-transduced VSMCs were exposed to CAWS. Compared with empty vector-transduced cells, Lenti-SPOCK2-transduced VSMCs exhibited significantly reduced protein expression levels of MMP2 following CAWS induction (**Figures 7A, B**). Furthermore, gelatin zymography was performed to assess MMP2 activity within VSMCs. The results demonstrated that CAWS induction significantly increased the levels of activated MMP2 in the vector-transduced VSMCs, whereas SPOCK2 overexpression significantly suppressed MMP2 activation, maintaining it in a relatively inactive state (**Figures 7C, D**).

**Figure 7:**
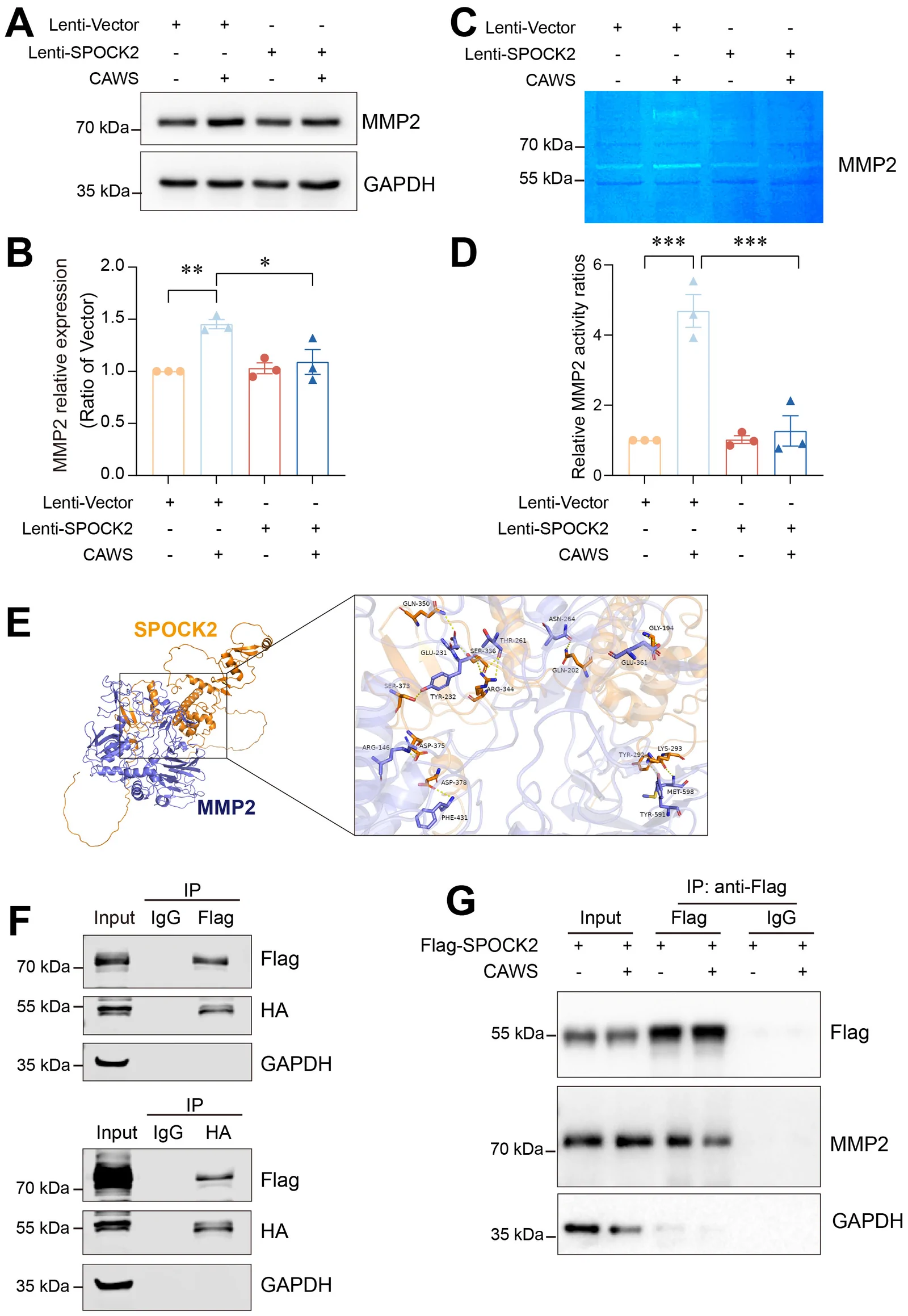
SPOCK2 modulated MMP2 activation via physical interaction in CAWS-induced VSMCs. **(A)** Representative Western immunoblots and **(B)** quantitative densitometric estimation of MMP2 protein levels in Lenti-Vector- and Lenti-SPOCK2-transduced VSMCs after CAWS induction (n=3 per group). **(C)** Representative gelatin zymography gel and **(D)** quantitative analysis showing MMP2 proteolytic activity (n=3 per group). **(E)** Overview of the three-dimensional (3D) macromolecular docking complex of SPOCK2-MMP2. Detailed ribbon diagram highlighting the specific interaction interface. **(F)** Co-immunoprecipitated (Co-IP) analysis of HEK 293T cells transiently co-transfected with Flag-tagged MMP2 and HA-tagged SPOCK2 (n=3). **(G)** Co-IP analysis of primary VSMCs stably transduced with Flag-tagged SPOCK2 lentivirus under CAWS-induced conditions (n=3). Data are represented as mean ± SEM. *P < 0.05, **P < 0.01, ***P < 0.001.

To visually verify the spatial relationship between SPOCK2 and MMP2 within the cellular compartment, dual-label immunofluorescence co-staining was performed in primary VSMCs *in vitro*. Confocal microscopy demonstrated that SPOCK2 and MMP2 co-localize within the cytoplasm (**Supplementary Figures 5A, B**), providing visual evidence that these two proteins occupy the same subcellular niches to potentially facilitate functional crosstalk.

To explore whether SPOCK2 interacted with MMP2, HEK293T cells were co-transfected with plasmids encoding Flag-tagged MMP2 and HA-tagged SPOCK2. Co-immunoprecipitation analysis strongly confirmed a physical interaction between Flag-tagged MMP2 and HA-tagged SPOCK2 (**Figure 7F**). To further elucidate the structural basis and thermodynamic feasibility of the physical interaction between SPOCK2 and MMP2, we performed *in silico* macromolecular docking simulation using HADDOCK. The predicted docking complex demonstrated high structural and geometric complementarity between the two proteins (**Figure 7E** and **Supplementary Table 2**). Subsequent interfacial analysis using the PDBePISA server revealed that the SPOCK2-MMP2 interaction interface was highly stable, yielding a robust thermodynamic binding free energy (△G) of -32.2 kcal/mol. This computational simulation strongly supports a spatial configuration wherein SPOCK2 predicts stable association with MMP2, providing a structural rationale for the steric hindrance that potentially obstructs MMP2 activation.

To further validate the interactions between SPOCK2 and MMP2 under CAWS-induced conditions in VSMCs, Flag-tagged-SPOCK2-overexpressing VSMCs were exposed to CAWS. The interaction between SPOCK2 and MMP2 was significantly diminished following CAWS induction (**Figure 7G**). These findings indicate that CAWS attenuates the interaction between SPOCK2 and MMP2 in VSMCs, thereby removing the inhibitory effect of SPOCK2 and enhancing MMP2 activity during CAWS-induced vasculitis.

## Discussion

Coronary artery aneurysm is one of the most severe complications of KD (15). Although IVIG therapy has significantly reduced the overall incidence of coronary artery aneurysms, the efficacy of IVIG in managing giant aneurysms remains limited (16). Even though the diameter of the aneurysmal artery is regressed following IVIG treatment, pathological remodeling of the vessel wall persists, which is a long-term risk for cardiovascular adverse events in KD patients (17). However, the precise molecular mechanisms underlying arterial aneurysm formation in KD remain poorly understood. Pathological remodeling of the vascular wall, characterized by ECM degradation and VSMC phenotype switch, represents the defining hallmark of coronary and aortic aneurysms in KD. Although the involvement of various MMPs in driving elastin breakdown has been documented, the upstream endogenous breaks that restrict this catastrophic cascade within the inflammatory microenvironment remain largely uncharacterized. In the present study, we identified the matricellular protein SPOCK2 as a novel suppressor of KD-associated vascular lesions. By leveraging clinical datasets, a CAWS-induced murine vasculitis model, and *in vitro* primary VSMC co-culture systems, we demonstrated that SPOCK2 expression is profoundly downregulated during acute KD. More importantly, AAV2/9-mediated or lentiviral overexpression of SPOCK2 successfully rescued the contractile phenotype of VSMCs, thereby mitigating vasculitis and preventing abdominal aortic aneurysm expansion. Mechanistically, our results unveil a novel biochemical axis wherein SPOCK2 physically binds to MMP2 and suppresses its enzymatic activation, serving as an essential checkpoint governing vascular wall homeostasis.

VSMCs are the most abundant cellular components in the arterial wall. The phenotypic plasticity of VSMCs allows them to transition from a quiescent, contractile state to a highly proliferative, migratory, and synthetic phenotype in response to vascular injury or persistent inflammation (18). Recent advances in single-cell RNA sequencing technology have revealed that synthetic VSMCs can further differentiate into various phenotypes, such as mesenchymal-like, fibroblast-like, macrophage-like, osteocyte-like, and adipocyte-like phenotypes (19). During acute KD vasculitis, this phenotypic switch often manifests as a transition toward a fibroblast-like or mesenchymal-like state, facilitating luminal myofibroblast proliferation (LMP) and structural weakening of the vessel wall (19–23). Zhang et al. reported that KD patients with giant coronary artery aneurysms exhibited a sustained VSMC dedifferentiation phenotype, characterized by decreased expression of VSMC contractile markers (*Acta2*, *Cnn1*, and *Tagln*) and increased levels of dedifferentiation markers (*Pcna*, *Opn*, and *Pdfgrβ*) (24).

Our current findings revealed a significant downregulation of *Acta2* mRNA expression in hCASMCs following exposure to KD patients’ serum. In contrast, the mRNA expression of *Mmp2* was significantly upregulated under the same conditions, indicating a potential change in VSMCs phenotype toward a synthetic, proliferative state. Moreover, our *in vivo* findings using the CAWS-induced murine vasculitis model clearly recapitulated this phenomenon, showing a stark loss of contractile markers (such as α-SMA and Tagln) alongside an abrupt upsurge in synthetic proteins (including periostin, vimentin and PCNA) in both the aortic root and abdominal aorta. Therefore, the phenotype switch of VSMCs to a fibroblast-like state plays a pivotal role in the pathogenesis of KD vasculitis by promoting vascular remodeling and inflammatory response.

SPOCK2 is a chimeric proteoglycan and a member of the SPARC-related matricellular protein family (25). However, only a few studies have investigated the role of SPOCK2 in the context of pulmonary diseases and cancer; and its functions and mechanisms in vascular disease remain poorly understood (26). Through unbiased analysis of SPARC family members in the transcriptomic profiling of KD cohorts and subsequent experimental validation, we observed a strong correlation between the loss of SPOCK2 and the onset of this pathogenic phenotype switch. The observation that SPOCK2 replenishment via viral vectors effectively promoted the recovery of VSMCs toward their contractile state highlights its important protective role in maintaining VSMC identity under inflammatory stress. Moreover, the role of SPOCK2 in regulating arterial structural stability suggests that targeting SPOCK2 may represent a potential strategy for mitigating vascular inflammatory responses and limiting lesion development in experimental models of KD.

Matrix metalloproteinases are a family of enzymes involved in the degradation and remodeling of ECM, influencing a wide range of physiological and pathological processes (27). Recent studies have demonstrated that MMP2 is a key therapeutic indicator in KD, showing a significant correlation with the development of coronary artery aneurysms and functioning as an important biomarker of coronary artery lesions in KD patients (28). Moreover, MMP2 has been identified to play a critical role in VSMC phenotype switch and arterial wall remodeling (29). Increasing evidence has also determined that MMP-2 is involved in the pathogenesis of aortic aneurysms (30, 31). In our study, MMP2 expression was significantly increased in CAWS-induced abdominal aortic aneurysms, which was attenuated by AAV2/9-mediated SPOCK2 overexpression. These findings suggest that SPOCK2 modulates the pathological process of aortic aneurysm formation in KD, at least in part, through regulating MMP2.

It is well-established that both MMP2 and MMP9 serve as pivotal pathological matrix-degrading enzymes during the acute vascular inflammation and structural remodeling characteristics of KD vasculitis (32–34). However, in our specific experimental model, we prioritized the evaluation of the MMP2 cascade over MMP9 for several compelling reasons. First, unbiased transcriptomic RNA-sequencing profiling of the abdominal aorta revealed that MMP2 expression was specifically and robustly suppressed following AAV2/9-SPOCK2 administration under CAWS challenge. Second, this focus is strongly supported by known upstream biochemical hierarchies. MMP2 is typically secreted in an inactive pro-enzymatic form, and its activation is regulated by the MT1-MMP-TIMP2 cell-surface complex, which serves as key regulatory co-factor for proteolytic processing (35). Previous biochemical studies have reported that SPOCK2 can bind to MT1-MMP via its SPARC extracellular domain to modulate this upstream MMP2 activation cascade (36). Crucially, our study expands this paradigm by demonstrating that SPOCK2 does not merely act through upstream co-activators but also engages in stable physical interaction with the substrate, MMP2 itself. Our Co-IP assays and macromolecular docking simulations reveal a novel spatial configuration wherein SPOCK2 anchors to MMP2, providing a structural rationale for steric hindrance that potentially obstructs its activation. While MMP9 remains an important player in the broader scope of KD pathogenesis, the above intersecting lines of evidence selectively identify the SPOCK2-MMP2 interaction axis as the principal functional cascade driving vascular wall homeostasis in this setting.

Although the involvement of SPOCK2 in regulating MMP2 has been reported in other diseases (31–33), its precise mechanisms in KD are not well understood. A particularly intriguing and crucial finding of our study is the physical and functional interaction between SPOCK2 and MMP2, which provides a fresh paradigm for understanding the regulation of ECM proteolysis. MMP2 is known to be secreted in an inactive pro-enzymatic form, and its activation is regulated by MT1-MMP-TIMP2 complex (34). Previous biochemical studies have reported that SPOCK2 can bind to MT1-MMP via its SPARC extracellular domain to modulate MMP2 activation in a rat model of chronic constriction injury (35). However, whether SPOCK2 engages the substrate MMP2, and what the functional consequences of such an engagement might be, have not been examined. Through Co-IP assay in both HEK293T cells and primary VSMCs, we confirmed that SPOCK2 robustly interacts with MMP2. Crucially, gelatin zymography revealed that instead of facilitating proteolysis, SPOCK2 overexpression markedly diminished the levels of active MMP2, keeping it sequestered in its inactive pro-form.

Our *in vivo* data further enriched this mechanistic landscape by demonstrating that SPOCK2 overexpression profoundly suppresses the local and systemic expression of inflammatory cytokines. This tissue-level anti-inflammatory effect may operate through a synergistic dual mechanism. First, at the cell-intrinsic level, preserving SPOCK2 prevents VSMCs from undergoing a synthetic phenotypic transition, thereby shutting down their hyper-secretory program and reducing direct VSMC-derived inflammatory cytokine production. Second, at the extracellular level, SPOCK2 safeguards ECM integrity by physically restricting MMP2 activation. Unchecked MMP2 activity digests structural elastin and collagen, releasing bioactive matrix fragments into the microenvironment. These fragments function as damage-associated molecular patterns (DAMPs) that bind to pattern recognition receptors on neighboring endothelial cells and infiltrating macrophages, significantly amplifying downstream NF-κB and cytokine signaling cascades. By physically interacting with MMP2 and limiting the generation of pro-inflammatory matrix fragments while helping maintain VSMCs in a quiescent state, SPOCK2 serves as an important regulatory checkpoint that helps disrupt this inflammatory amplification loop. Thus, SPOCK2 represents a multi-functional therapeutic target that simultaneously dampens inflammation and stabilizes the vascular architecture.

A key question arising from the use of diverse KD animal models is how distinct microbial stimuli triggers vascular lesion formation. While CAWS predominantly activates Dectin-2/Syk signaling via fungal β-glucan and mannan components, LCWE initiates vasculitis primarily through TLR2/MyD88 pathways engaged by bacterial cell-wall peptidoglycans (37). Despite these divergent upstream pattern-recognition receptor (PRR) triggers, our findings demonstrate that SPOCK2 depletion occurs consistently across abdominal aortic wall remodeling in both models. This suggests that SPOCK2 suppression is not restricted to a specific fungal PRR cascade but rather represents a convergent downstream node in vascular inflammation. Delineating the precise contribution of isolated microbial ligands to SPOCK2 transcriptional regulation represents an important avenue for future immunobiological studies.

We acknowledge that a limitation of our *in vivo* rescue experiments is the use of a global AAV2/9 vector administered systemically, which does not restrict SPOCK2 overexpression exclusively to the smooth muscle compartment. While our *in vitro* transwell assays isolate a direct, cell-intrinsic protective role for SPOCK2 within primary VSMCs, the *in vivo* attenuation of vasculitis and aneurysm expansion may involve contributions from overexpressed SPOCK2 in neighboring endothelial cells or circulating immune cells. Future studies utilizing smooth muscle-specific promoters or conditional knockout mouse strains will be necessary to precisely isolate the cell-type-specific physiological contributions of endogenous SPOCK2. Second, translational extrapolations from our animal model to human clinical disease warrant cautious interpretation. While our human *in vitro* studies utilized hCASMCs and patient-derived blood samples, *in vivo* protective efficacy was evaluated in a murine model of vasculitis, where pathological vascular expansion predominantly occurs in the abdominal aorta. Although human KD predominantly affects the coronary vasculature, murine models inherently exhibit structural and hemodynamic differences, with aortic lesions serving as the standard surrogate for severe vascular inflammation and arterial remodeling. Future investigations utilizing coronary-focused preclinical models or advanced tissue-engineered human vessel constructs will be valuable to further confirm the coronary-specific therapeutic window of SPOCK2.

In conclusion, our study demonstrates that SPOCK2 is an important endogenous protector against vascular remodeling in KD. By physically associating with MMP2 and inhibiting its activation, SPOCK2 prevents the pathogenic switch of VSMCs toward a fibroblast-like phenotype. Restoring SPOCK2 expression represents a promising preventive strategy for stabilizing vascular integrity and mitigating severe arterial wall remodeling associated with Kawasaki disease.

## Data Availability

The datasets presented in this study can be found in online repositories. The names of the repository/repositories and accession number(s) can be found below: https://www.cncb.ac.cn/, CRA029797.
