# Supplementary material for: SPOCK2-mediated phenotype switch of vascular smooth muscle cells contributes to aortic aneurysm in Kawasaki disease
Source: Front Immunol. 2026 Oct 23;17:1902009. doi: 10.3389/fimmu.2026.1902009 (PMC13739779; doi:10.3389/fimmu.2026.1902009)
Supplement: Supplementary file 1 [file DataSheet1.pdf]

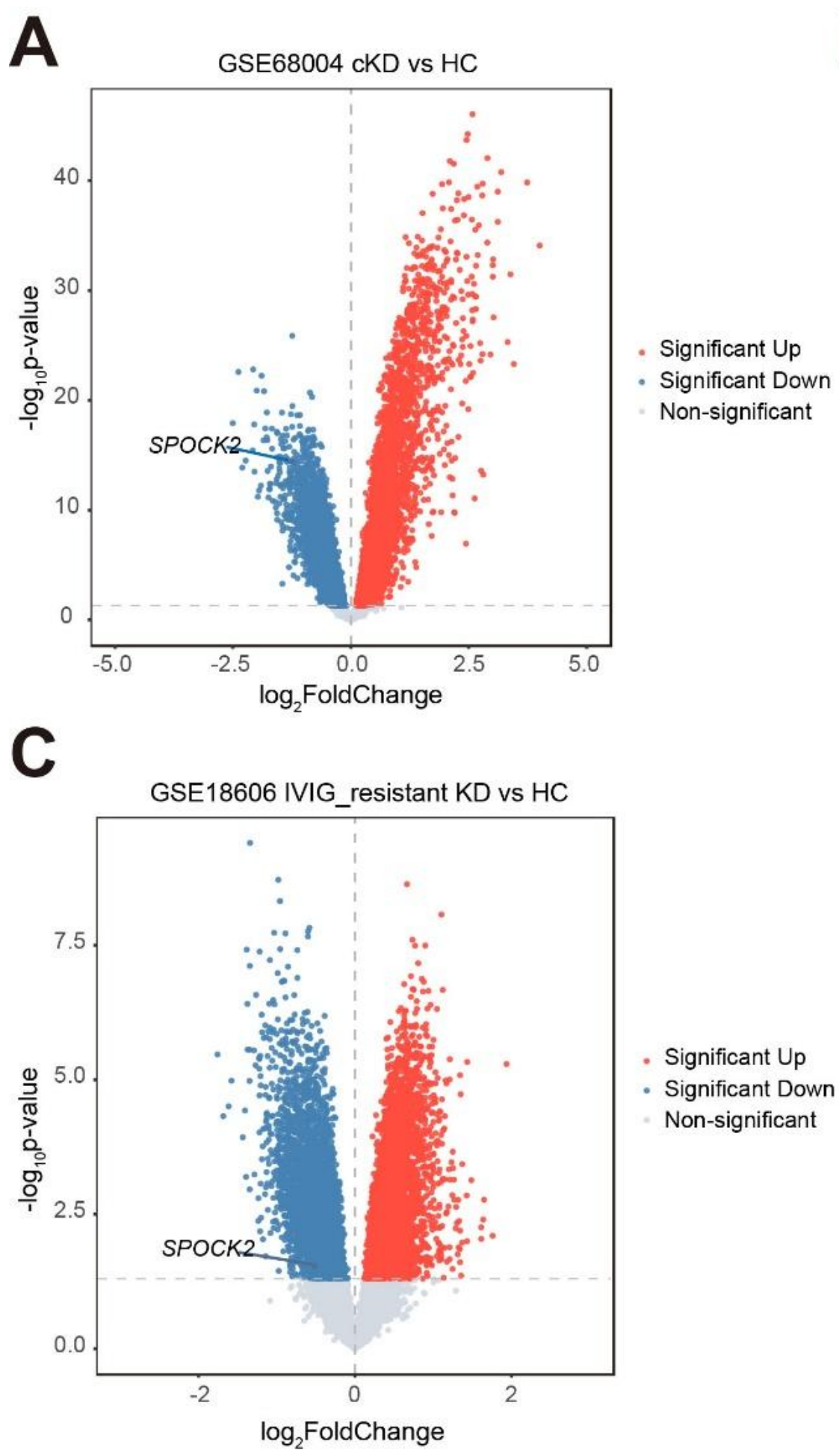

Supplementary Figure 1 Transcriptional profiles of *SPOCK2* in peripheral blood of KD patients. Volcano plot analysis of differentially expressed genes (DEGs) in human peripheral blood of health control compared with KD patients in GSE68004 (A) and GSE73461 datasets (B). (C) Volcano plot analysis of DEGs in human peripheral blood of healthy control compared with IVIG-resistant KD patients in GSE18606 datasets. Shown significant upregulated DEGs in red and downregulated DEGs in blue. DEGs were defined as $|\log_2 FC| > 1$and p-value < 0.05.

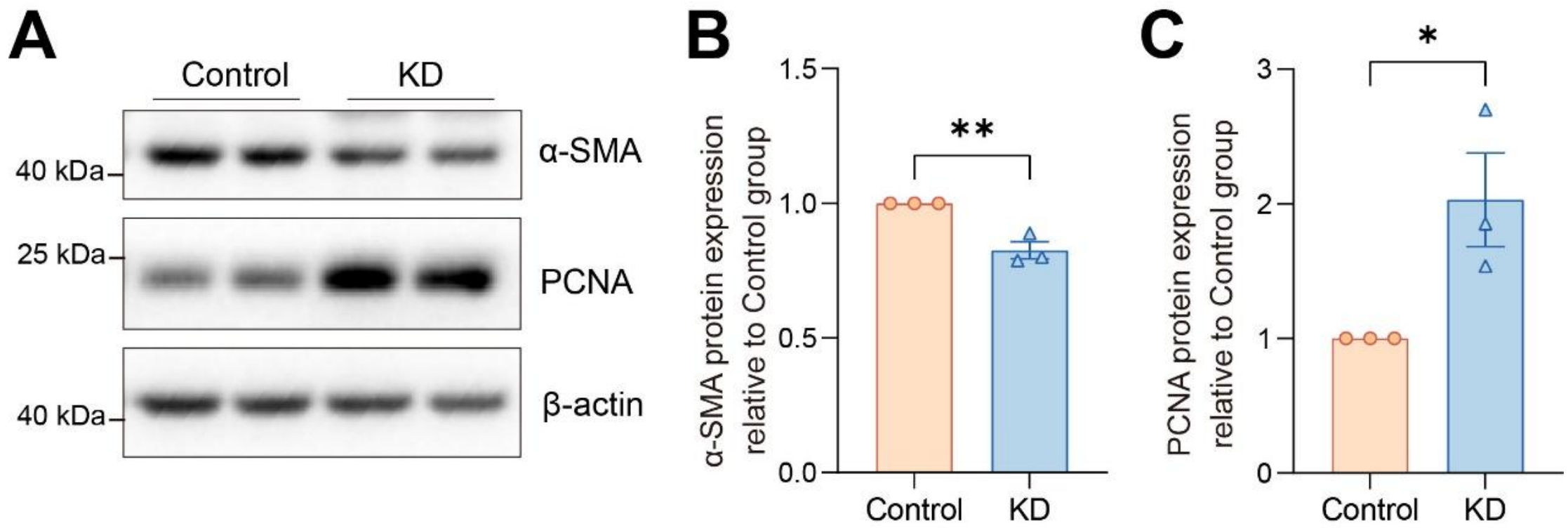

Supplementary Figure 2 Phenotypic switch of human coronary artery smooth muscle cells induced by serum from KD patients. Representative Western blot of a-SMA and PCNA (A) in human coronary artery smooth muscle cells (hCASMCs) treated with serum from health control or KD patients (n=3). Quantitative data shown in B, C. Data are represented as mean ± SEM. * $p < 0.05$, ** $p < 0.01$.

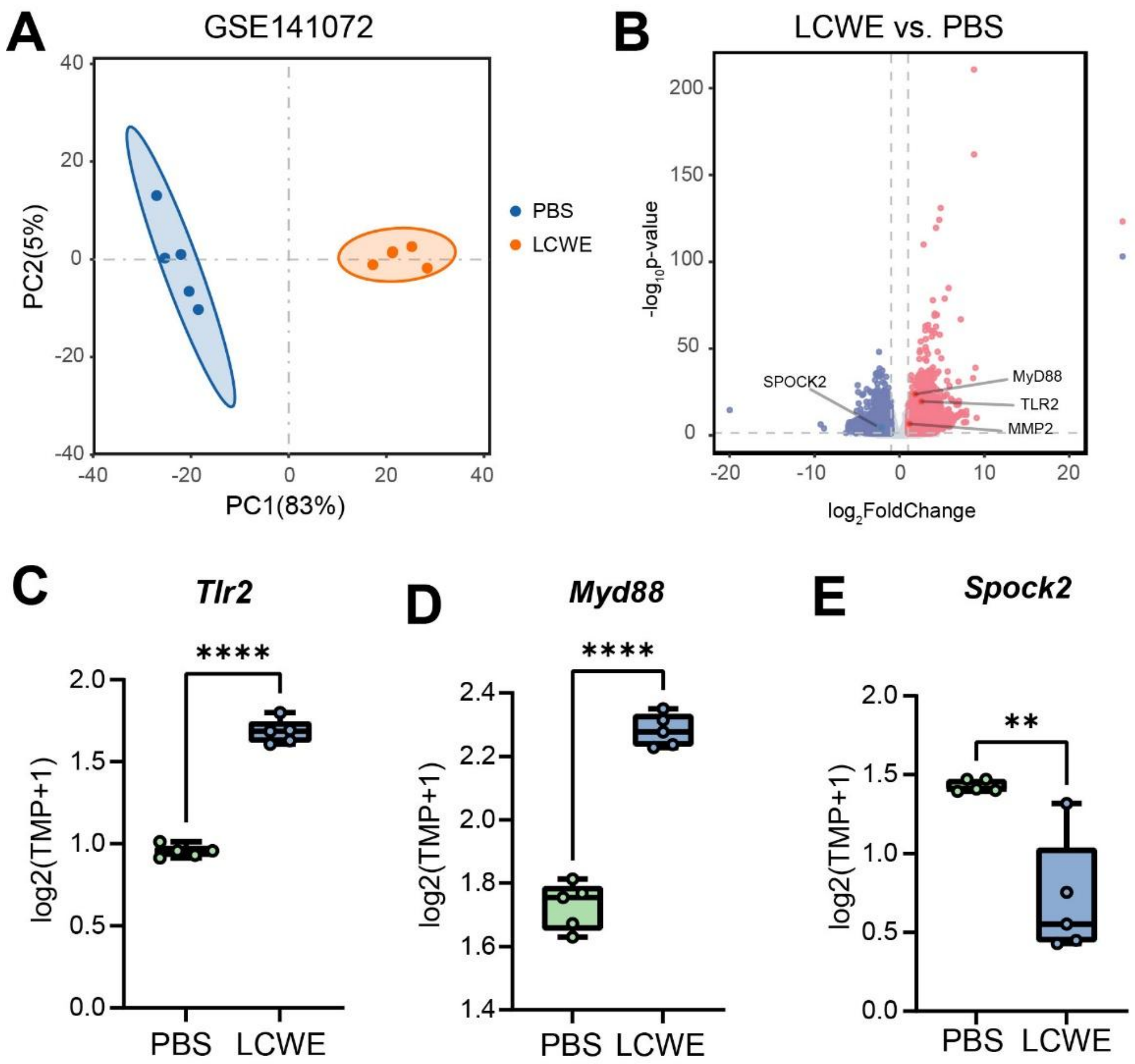

Supplementary Figure 3 RNA-seq analysis of the abdominal aorta from LCWE-induced murine vasculitis. (A) PCA plots illustrate the differences in transcriptional profiles of the abdominal aorta between PBS and LCWE-injected mice from GEO dataset GSE141072 (male mice, n=5 per group). (B) Volcano plot revealed the up-regulated and down-regulated DEGs in the transcriptional profiles of the abdominal aorta from PBS and LCWE-injected mice from GEO dataset GSE141072 ($|log_2FC|>2$, adjusted *p*-value <0.05). (C-E) Relative expressions of *Tlr2*, *Myd88*, and *Spock2* in abdominal aorta from PBS and LCWE-injected mice from GEO dataset GSE141072 (male mice, n=5 per group). ** $p < 0.01$, ****$p < 0.0001$.

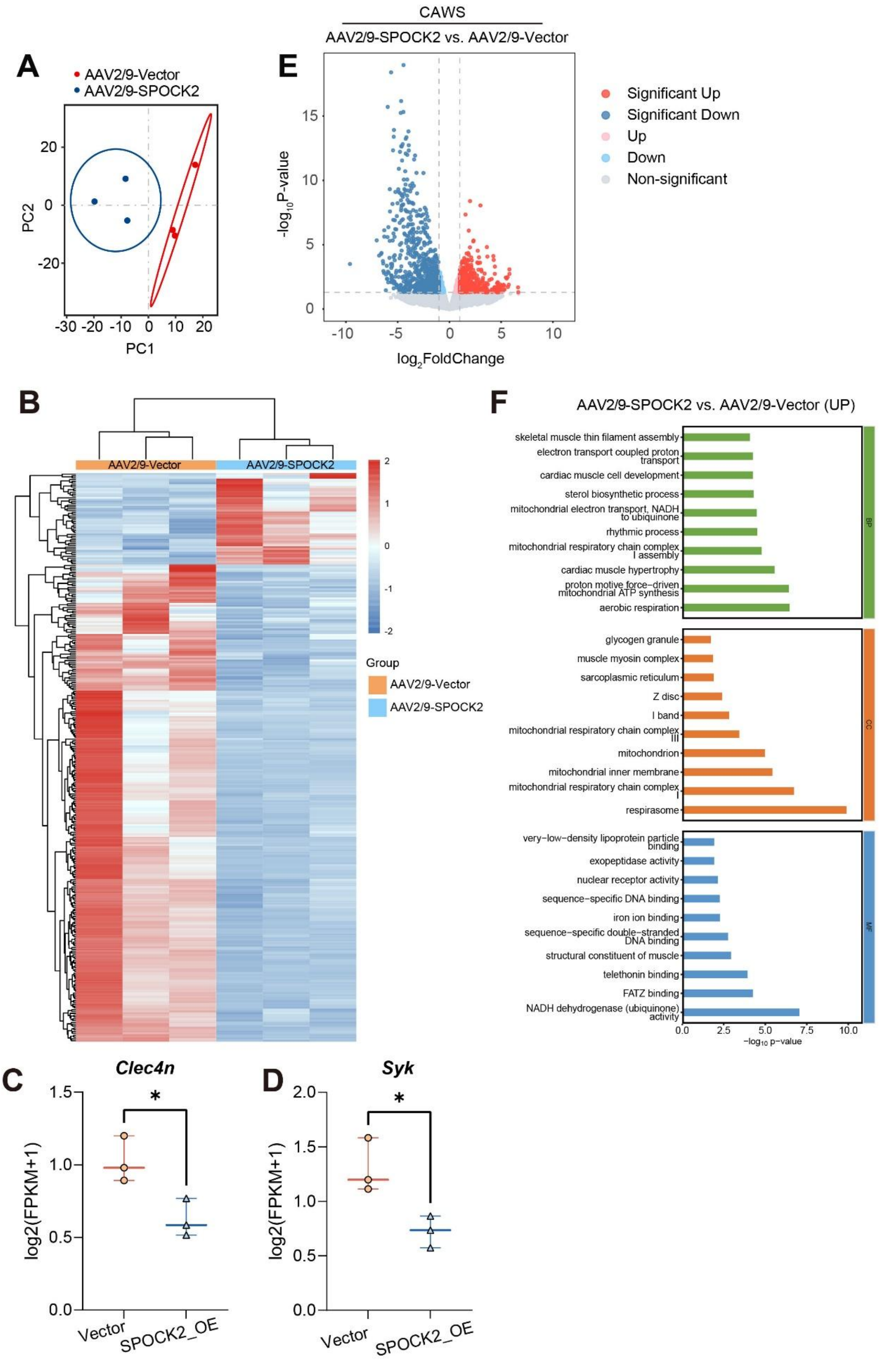

Supplementary Figure 4 RNA-seq analysis of the abdominal aorta from AAV2/9-Vector-injected and AAV2/9-SPOCK2-injected mice at 28 days post CAWS induction. (A) PCA plots illustrate the differences in transcriptional profiles of the abdominal aorta

between AAV2/9-Vector-injected and AAV2/9-SPOCK2-injected mice 28 days after CAWS induction (n=3 per group). (B) Heatmap displays the transcriptional profiles of the abdominal aorta in AAV2/9-Vector-injected and AAV2/9-SPOCK2-injected mice at 28 days after CAWS induction (n=3 per group). (C-D) Relative expression levels of *Clec4n* and *Syk* in AAV2/9-Vector-injected (Vector) and AAV2/9-SPOCK2-injected (SPOCK2_OE) mice at 28 days after CAWS induction (n=3 per group). (E) Volcano plot diagram revealed the up-regulated and down-regulated DEGs in the transcriptional profiles of the abdominal aorta from AAV2/9-Vector-injected and AAV-SPOCK2-injected mice at 28 days after CAWS induction ($|\log_2 FC| > 2$, adjusted p-value < 0.05) (n=3 per group). (F) Gene Ontology (GO) enrichment analysis identified the top 10 upregulated GO terms separately for biological processes (BP), cell components (CC), and molecular function (MF) (n=3 per group). * $p < 0.05$.

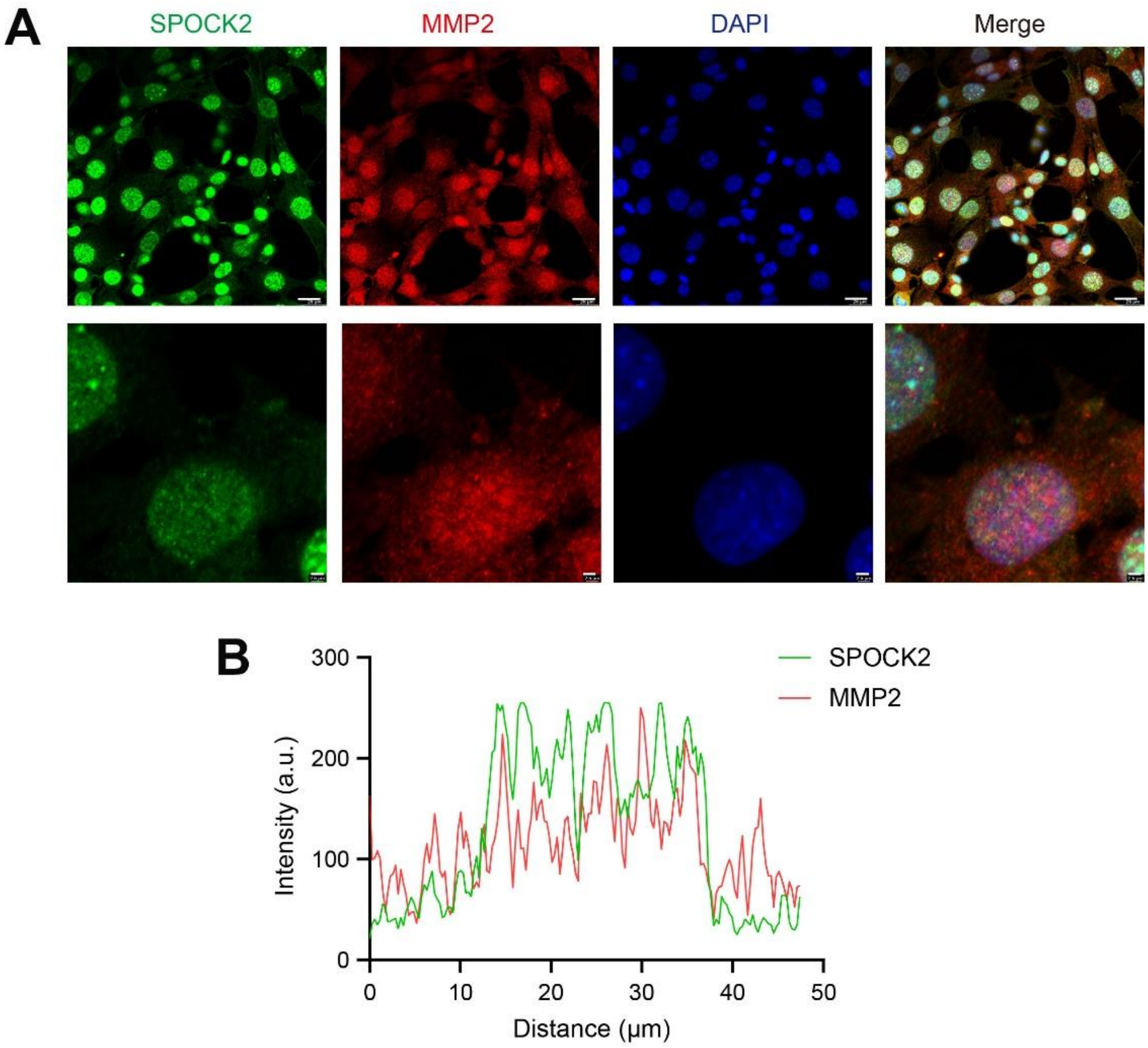

Supplementary Figure 5 Dual immunofluorescence co-localization and quantitative analysis of SPOCK2 and MMP2 in primary VSMCs. (A) Representative confocal micrographs showing dual-label immunofluorescence staining for SPOCK2 (green) and MMP2 (red) in VSMCs. Cell nuclei were counterstained with DAPI (blue). High-magnification merged panels demonstrate marked SPOCK2 and MMP2 under baseline conditions. Scale bars (upper panels): 20 μm; scale bars (lower panels): 2.5 μm. (B) Semi-quantitative analysis of SPOCK2 and MMP2 co-localization immunofluorescence intensity. Data are presented as Mean Fluorescence Intensity in arbitrary units (a.u.).

Supplementary Table 1. Primers used for qPCR.

Gene name	species	Sequence
Acta2	*Mus musculus*	Forward: 5’ GACTCACAACGTGCCTATCT 3’<br>Reverse: 5’ CTCACGCTCGGCAGTAGTC 3’
Myh11	*Mus musculus*	Forward: 5’ CCTCACCAGGAGTCAACCAC 3’<br>Reverse: 5’ GGTCAGGGAAAGGTTGGGAG 3’
Tagln	*Mus musculus*	Forward: 5’ TGGTTTATGAAGAAAGCCCAGGA 3’<br>Reverse: 5’ GAAGGCCAATGACGTGCTTC 3’
Lum1	*Mus musculus*	Forward: 5’ AAAGAGGATGCTGTCTCGGC 3’<br>Reverse: 5’ TGTAGGTAGACCAGCAGGCA3’
Fn1	*Mus musculus*	Forward: 5’ GCTCAGCAAATCGTGCAGC3’<br>Reverse: 5’ CTAGGTAGGTCCGTTCCCACT3’
Il1	*Mus musculus*	Forward: 5’ GCAACTGTTCCTGAACTCAACT3’<br>Reverse: 5’ ATCTTTTGGGGTCCGTCAACT3’
Il6	*Mus musculus*	Forward: 5’ CTGCAAGAGACTTCCATCCAG3’<br>Reverse: 5’ AGTGGTATAGACAGGTCTGTTGG3’
Nos2	*Mus musculus*	Forward: 5’ GGAGTGACGGCAAACATGACT3’<br>Reverse: 5’ TCGATGCACAACTGGGTGAAC3’
Mmp2	*Mus musculus*	Forward: 5’ CCTGGACCCTGAAACCGTG3’<br>Reverse: 5’ TCCCCATCATGGATTCGAGAA3’
Mmp9	*Mus musculus*	Forward: 5’ GCGTCGTGATCCCCACTTAC3’<br>Reverse:5’ CAGGCCGAATAGGAGCGTC3’
Spock2	*Mus musculus*	Forward: 5’ ATGGCTGTCGTCCATCTCGC3’<br>Reverse:5’ TTGCCTCCGTGGAGTTTCAG3’
Clec4n	*Mus musculus*	Forward: 5’ CCTGTTTCATTGCGAGCTGTG3’<br>Reverse: 5’ ACTGAAGCAGGTGAGACTGGAA3’
Syk	*Mus musculus*	Forward: 5’ TCCAGTAAGAGTGACGTTTGGAG3’<br>Reverse: 5’ACATCTCTCTCGGGCATCCT3’
Gapdh	*Mus musculus*	Forward: 5’ AGGTCGGTGTGAACGGATTTG3’<br>Reverse:5’ TGTAGACCATGTAGTTGAGGTCA3’
ACTA2	*Homo Sapiens*	Forward: 5’ GATGGTGGGAATGGGACAAA3’<br>Reverse:5’ GCCATGTTCTATCGGGTACTTC3’
MMP2	*Homo Sapiens*	Forward: 5’ GATACCCCTTTGACGGTAAGGA3’<br>Reverse:5’ CCTTCTCCCAAGGTCCATAGC3’
GAPDH	*Homo Sapiens*	Forward: 5’ GGCAAATTCCATGGCACCGT 3’<br>Reverse:5’ GGGATCTCGCTCCTGGAAGA 3’

Supplementary Table 2. Results of molecular docking for hydrogen-bonding interaction in SPOCK2-MMP2 complex.

SPOCK2 Receptor Residue	MMP2 Ligand Residue	Interaction Constituents	Distance (Å)
A: LYS195	C: GLU361	A: LYS195: N-C: GLU361:OE2	3.74
A: LYS293	C: TYR591	A: LYS293:NZ-C: TYR591: OH	2.57
A: GLN321	C: LYS267	A: GLN321:NE2-C: LYS267:O	3.89
A: GLN321	C: ASP268	A: GLN321:NE2-C: ASP268:O	3.63
A: ARG344	C: TYR232	A: ARG344:NH1-C: TYR232:O	3.56
A: ARG344	C: THR261	A: ARG344:NH2-C: THR261:O	3.45
A: GLN350	C: GLU231	A: GLN350:NE2-C: GLU231:OE1	3.15
A: SER373	C: TYR232	A: SER373: OG-C: TYR232: OH	2.74
A: ASP375	C: ASP142	A: ASP375: N-C:ASP142-OD1	3.33
A: THR291	C: LEU561	A: THR291:O-C: LEU561: N	2.89
A: TYR292	C: MET598	A: TYR292:O-C: MET598: N	2.64
A: LYS293	C: MET598	A: LYS293:O-C: MET598: N	3.39
A: ASP377	C: ASN430	A: ASP377:O-C: ASN430:ND2	3.71
